# A Multilaboratory Comparison of Calibration Accuracy and the Performance of External References in Analytical Ultracentrifugation

**DOI:** 10.1371/journal.pone.0126420

**Published:** 2015-05-21

**Authors:** Huaying Zhao, Rodolfo Ghirlando, Carlos Alfonso, Fumio Arisaka, Ilan Attali, David L. Bain, Marina M. Bakhtina, Donald F. Becker, Gregory J. Bedwell, Ahmet Bekdemir, Tabot M. D. Besong, Catherine Birck, Chad A. Brautigam, William Brennerman, Olwyn Byron, Agnieszka Bzowska, Jonathan B. Chaires, Catherine T. Chaton, Helmut Cölfen, Keith D. Connaghan, Kimberly A. Crowley, Ute Curth, Tina Daviter, William L. Dean, Ana I. Díez, Christine Ebel, Debra M. Eckert, Leslie E. Eisele, Edward Eisenstein, Patrick England, Carlos Escalante, Jeffrey A. Fagan, Robert Fairman, Ron M. Finn, Wolfgang Fischle, José García de la Torre, Jayesh Gor, Henning Gustafsson, Damien Hall, Stephen E. Harding, José G. Hernández Cifre, Andrew B. Herr, Elizabeth E. Howell, Richard S. Isaac, Shu-Chuan Jao, Davis Jose, Soon-Jong Kim, Bashkim Kokona, Jack A. Kornblatt, Dalibor Kosek, Elena Krayukhina, Daniel Krzizike, Eric A. Kusznir, Hyewon Kwon, Adam Larson, Thomas M. Laue, Aline Le Roy, Andrew P. Leech, Hauke Lilie, Karolin Luger, Juan R. Luque-Ortega, Jia Ma, Carrie A. May, Ernest L. Maynard, Anna Modrak-Wojcik, Yee-Foong Mok, Norbert Mücke, Luitgard Nagel-Steger, Geeta J. Narlikar, Masanori Noda, Amanda Nourse, Tomas Obsil, Chad K. Park, Jin-Ku Park, Peter D. Pawelek, Erby E. Perdue, Stephen J. Perkins, Matthew A. Perugini, Craig L. Peterson, Martin G. Peverelli, Grzegorz Piszczek, Gali Prag, Peter E. Prevelige, Bertrand D. E. Raynal, Lenka Rezabkova, Klaus Richter, Alison E. Ringel, Rose Rosenberg, Arthur J. Rowe, Arne C. Rufer, David J. Scott, Javier G. Seravalli, Alexandra S. Solovyova, Renjie Song, David Staunton, Caitlin Stoddard, Katherine Stott, Holger M. Strauss, Werner W. Streicher, John P. Sumida, Sarah G. Swygert, Roman H. Szczepanowski, Ingrid Tessmer, Ronald T. Toth, Ashutosh Tripathy, Susumu Uchiyama, Stephan F. W. Uebel, Satoru Unzai, Anna Vitlin Gruber, Peter H. von Hippel, Christine Wandrey, Szu-Huan Wang, Steven E. Weitzel, Beata Wielgus-Kutrowska, Cynthia Wolberger, Martin Wolff, Edward Wright, Yu-Sung Wu, Jacinta M. Wubben, Peter Schuck

**Affiliations:** 1 Dynamics of Macromolecular Assembly Section, Laboratory of Cellular Imaging and Macromolecular Biophysics, National Institute of Biomedical Imaging and Bioengineering, National Institutes of Health, Bethesda, Maryland, 20892, United States of America; 2 Laboratory of Molecular Biology, National Institute of Diabetes and Digestive and Kidney Diseases, National Institutes of Health, Bethesda, Maryland, 20892, United States of America; 3 Analytical Ultracentrifugacion and Light Scattering Facility, Centro de Investigaciones Biológicas, CSIC, Madrid, 28040, Spain; 4 Life Science Research Center, Nihon University, College of Bioresource Science, Fujisawa, 252–0880, Japan; 5 Department of Biochemistry and Molecular Biology, Tel Aviv University, Tel Aviv, 69978, Israel; 6 Department of Pharmaceutical Sciences, University of Colorado Denver Anschutz Medical Campus, Aurora, Colorado, 80045, United States of America; 7 Department of Chemistry and Biochemistry, Center for Retrovirus Research, and Center for RNA Biology, The Ohio State University, Columbus, Ohio, 43210, United States of America; 8 Redox Biology Center, University of Nebraska-Lincoln, Lincoln, Nebraska, 68588, United States of America; 9 Department of Microbiology, University of Alabama at Birmingham, Birmingham, Alabama, 35294, United States of America; 10 Supramolecular Nanomaterials and Interfaces Laboratory, Institute of Materials, École Polytechnique Fédérale de Lausanne, Lausanne, CH-1015, Switzerland; 11 National Centre for Macromolecular Hydrodynamics, University of Nottingham, School of Biosciences, Sutton Bonington, LE12 5RD, United Kingdom; 12 Structural Biology Platform IGBMC, Illkirch, 67400, France; 13 Department of Biophysics, The University of Texas Southwestern Medical Center, Dallas, Texas, 75390, United States of America; 14 Beckman Coulter, Inc., Life Science Division, Indianapolis, Indiana, 46268, United States of America; 15 School of Life Sciences, University of Glasgow, Glasgow, G37TT, United Kingdom; 16 Division of Biophysics, Institute of Experimental Physics, Faculty of Physics, University of Warsaw, Warsaw, 02–089, Poland; 17 JG Brown Cancer Center, University of Louisville, Louisville, Kentucky, 40202, United States of America; 18 Department of Molecular Genetics, Biochemistry and Microbiology, University of Cincinnati College of Medicine, Cincinnati, Ohio, 45267, United States of America; 19 Physical Chemistry, University of Konstanz, 78457, Konstanz, Germany; 20 Program in Molecular Medicine, University of Massachusetts Medical School, Worcester, Massachusetts, 01605, United States of America; 21 Institute for Biophysical Chemistry, Hannover Medical School, 30625, Hannover, Germany; 22 Institute of Structural and Molecular Biology Biophysics Centre, Birkbeck, University of London and University College London, London, WC1E 7HX, United Kingdom; 23 Department of Physical Chemistry, University of Murcia, Murcia, 30071, Spain; 24 Univ. Grenoble Alpes, IBS, F-38044, Grenoble, France; 25 CNRS, IBS, F-38044, Grenoble, France; 26 CEA, IBS, F-38044, Grenoble, France; 27 Protein Interactions Core, Department of Biochemistry, University of Utah School of Medicine, Salt Lake City, Utah, 84112, United States of America; 28 Wadsworth Center, New York State Department of Health, Albany, New York, 12208, United States of America; 29 Institute for Bioscience and Biotechnology Research, Fischell Department of Bioengineering, University of Maryland, Rockville, Maryland, 20850, United States of America; 30 Institut Pasteur, Centre of Biophysics of Macromolecules and Their Interactions, Paris, 75724, France; 31 Department of Physiology and Biophysics, School of Medicine, Virginia Commonwealth University, Richmond, Virginia, 23220, United States of America; 32 Materials Science and Engineering Laboratory, National Institute of Standards and Technology, Gaithersburg, Maryland, 20899, United States of America; 33 Department of Biology, Haverford College, Haverford, Pennsylvania, 19041, United States of America; 34 Laboratory of Chromatin Biochemistry, Max Planck Institute for Biophysical Chemistry, 37077, Göttingen, Germany; 35 Department of Structural and Molecular Biology, Darwin Building, University College London, London, WC1E 6BT, United Kingdom; 36 Diabetes Biophysics, Novo Nordisk A/S, Måløv, 2760, Denmark; 37 Research School of Chemistry, Section on Biological Chemistry, The Australian National University, Acton, ACT 0200, Australia; 38 Biochemistry, Cell and Molecular Biology Department, University of Tennessee, Knoxville, Tennessee, 37996–0840, United States of America; 39 Department of Biochemistry and Biophysics, University of California San Francisco, San Francisco, California, 94158, United States of America; 40 Tetrad Graduate Program, University of California San Francisco, San Francisco, California, 94158, United States of America; 41 Institute of Biological Chemistry, Academia Sinica, Taipei, 115, Taiwan; 42 Biophysics Core Facility, Scientific Instrument Center, Academia Sinica, Taipei, 115, Taiwan; 43 Institute of Molecular Biology and Department of Chemistry and Biochemistry, University of Oregon, Eugene, Oregon, 97403, United States of America; 44 Department of Chemistry, Mokpo National University, Muan, 534–729, Korea; 45 Enzyme Research Group, Concordia University, Montreal, Quebec, H4B 1R6, Canada; 46 Department of Physical and Macromolecular Chemistry, Faculty of Science, Charles University in Prague, Prague, 12843, Czech Republic; 47 Department of Biotechnology, Graduate School of Engineering, Osaka University, Suita, Osaka, 565–0871, Japan; 48 Department of Biochemistry and Molecular Biology, Colorado State University, Fort Collins, Colorado, 80523, United States of America; 49 Pharma Research and Early Development, Roche Innovation Center Basel, F. Hoffmann-LaRoche Ltd., Basel, 4070, Switzerland; 50 Analytical Biopharmacy Core, University of Washington, Seattle, Washington, 98195, United States of America; 51 Department of Biochemistry, University of New Hampshire, Durham, New Hampshire, 03824, United States of America; 52 Technology Facility, Department of Biology, University of York, York, YO10 5DD, United Kingdom; 53 Institute of Biochemistry and Biotechnology, Martin-Luther University Halle-Wittenberg, 06120, Halle, Germany; 54 Department of Biochemistry, Uniformed Services University of the Health Sciences, Bethesda, Maryland, 20814, United States of America; 55 Department of Biochemistry and Molecular Biology, Bio21 Instute of Molecular Science and Biotechnology, University of Melbourne, Parkville, 3010, Victoria, Australia; 56 Biophysics of Macromolecules, German Cancer Research Center, Heidelberg, 69120, Germany; 57 ICS-6, Structural Biochemistry, Research Center Juelich, 52428, Juelich, Germany; 58 Molecular Interaction Analysis Shared Resource, St. Jude Children’s Research Hospital, Memphis, Tennessee, 38105, United States of America; 59 Analytical Biophysics & Materials Characterization, Department of Chemistry and Biochemistry, University of Arizona, Tucson, Arizona, 85721, United States of America; 60 Central Instrument Center, Mokpo National University, Muan, 534–729, Korea; 61 Department of Chemistry and Biochemistry, Concordia University, Montreal, Quebec, H4B 1R6, Canada; 62 Department of Biochemistry, La Trobe Institute for Molecular Science, La Trobe University, Melbourne, Victoria, 3086, Australia; 63 Biochemistry and Biophysics Center, National Heart, Lung, and Blood Institute, National Institutes of Health, Bethesda, Maryland, 20892, United States of America; 64 Laboratory of Biomolecular Research, Department of Biology and Chemistry, Paul Scherrer Institute, 5232, Villigen PSI, Switzerland; 65 Department of Chemistry and Center for Integrated Protein Science, Technische Universität München, 85748, Garching, Germany; 66 Department of Biophysics and Biophysical Chemistry, Johns Hopkins University School of Medicine, Baltimore, Maryland, 21205, United States of America; 67 Research Complex at Harwell, Rutherford Appleton Laboratory, Oxfordshire, OX11 0FA, United Kingdom; 68 Proteome and Protein Analysis, University of Newcastle, Newcastle upon Tyne, NE1 7RU, United Kingdom; 69 Molecular Biophysics Suite, Department of Biochemistry, Oxford, Oxon, OX1 3QU, United Kingdom; 70 Biochemistry Department, University of Cambridge, Cambridge, CB2 1GA, United Kingdom; 71 Protein Function and Interactions, Novo Nordisk Foundation Center for Protein Research, Copenhagen, 2200, Denmark; 72 Core Facility, International Institute of Molecular and Cell Biology, Warsaw, 02–109, Poland; 73 Rudolf Virchow Center for Experimental Biomedicine, University of Würzburg, 97080, Würzburg, Germany; 74 Macromolecule and Vaccine Stabilization Center, University of Kansas, Lawrence, Kansas, 66047, United States of America; 75 Department of Biochemistry and Biophysics, University of North Carolina at Chapel Hill, Chapel Hill, North Carolina, 27599, United States of America; 76 Biochemistry Core Facility, Max Planck Institute of Biochemistry, 82152, Martinsried, Germany; 77 Drug Design Laboratory, Graduate School of Medical Life Science, Yokohama City University, Yokohama, 230–0045, Japan; 78 Laboratoire de Médecine Régénérative et de Pharmacobiologie, Ecole Polytechnique Fédérale de Lausanne, Lausanne, CH-1015, Switzerland; 79 Department of Chemical & Biomolecular Engineering, University of Delaware, Newark, Delaware, 19716, United States of America; German Cancer Research Center, GERMANY

## Abstract

Analytical ultracentrifugation (AUC) is a first principles based method to determine absolute sedimentation coefficients and buoyant molar masses of macromolecules and their complexes, reporting on their size and shape in free solution. The purpose of this multi-laboratory study was to establish the precision and accuracy of basic data dimensions in AUC and validate previously proposed calibration techniques. Three kits of AUC cell assemblies containing radial and temperature calibration tools and a bovine serum albumin (BSA) reference sample were shared among 67 laboratories, generating 129 comprehensive data sets. These allowed for an assessment of many parameters of instrument performance, including accuracy of the reported scan time after the start of centrifugation, the accuracy of the temperature calibration, and the accuracy of the radial magnification. The range of sedimentation coefficients obtained for BSA monomer in different instruments and using different optical systems was from 3.655 S to 4.949 S, with a mean and standard deviation of (4.304 ± 0.188) S (4.4%). After the combined application of correction factors derived from the external calibration references for elapsed time, scan velocity, temperature, and radial magnification, the range of *s*-values was reduced 7-fold with a mean of 4.325 S and a 6-fold reduced standard deviation of ± 0.030 S (0.7%). In addition, the large data set provided an opportunity to determine the instrument-to-instrument variation of the absolute radial positions reported in the scan files, the precision of photometric or refractometric signal magnitudes, and the precision of the calculated apparent molar mass of BSA monomer and the fraction of BSA dimers. These results highlight the necessity and effectiveness of independent calibration of basic AUC data dimensions for reliable quantitative studies.

## Introduction

As a first-principles based technique of physical chemistry, analytical ultracentrifugation (AUC) is capable of measuring absolute size and shape of particles, such as macromolecules and their complexes, free in solution [1]. The quantitative accuracy of physical parameters has been at the heart of the many advances facilitated by AUC throughout the last century. In the last few decades, progress in theory and computational analysis have afforded significantly enhanced resolution and precision of the derived macromolecular parameters, especially in sedimentation velocity (SV) analysis [2]. This is met by rapid developments of applications in different fields for which the quantitative aspect of AUC is critical, which include: improved capabilities of structure-based predictions of macromolecular shapes using hydrodynamic theory [3,4], as applied, for example, in the context of sophisticated data analysis in scattering techniques [5–7]; significant advances in techniques for studying membrane proteins by AUC [8]; and emerging applications in the characterization of nanoparticles [9,10] and biotechnology [11–17]. This is accompanied by a greatly extended concentration range for the application of AUC by fluorescence detection [18–20]. Thus, as the quantitative interpretation of sedimentation parameters is gaining in detail and applications, it is important to verify the basic accuracy of the AUC measurements. With view of biopharmaceutical applications of AUC, a number of recent studies have explored limits of detection and quantitation for trace aggregates of proteins [13,14,16,21–23], though not questioned the accuracy of basic data dimensions and sedimentation coefficients of the main components.

The question of calibration accuracy in AUC is not trivial and has a long history. Generally, the repeatability of *s*-values in the same instrument, as well as the reproducibility across different instruments and laboratories is considered to be better than 1% [24–26], and side-by-side measurements of samples in different rotor positions in the same run achieve an even higher precision of up to ≈0.1% in current instruments [25,27]. Unfortunately, these numbers do not bear on the accuracy. Due to the fact that systematic errors are not observable from repeat measurements in the same instrument (or even the same kind of instrument in the same or in another laboratory), systematic errors in any physical measurement are usually underestimated [28]. Yet, they may be the dominant source of error. For example, systematic discrepancies by as much as 10% between *s*-values measured in Svedberg’s oil turbine centrifuge and the early Spinco Model E were found and traced to errors of 4–5°C in the temperature calibration of the former [24,26,29,30]. The consequences of an unrecognized 10% error in s-values on their interpretation are drastic: for example, the 30S subunit of the 70S ribosome would appear to be a 33S subunit of a novel 77S particle; axial ratios estimated from hydrodynamic friction may appear to be 30% too small [31]; and even stronger error amplification can occur in the calculation of particle composition from *s*-values. This highlights the need for independent control experiments in ascertaining the true accuracy of AUC parameters, to detect calibration errors in basic data dimensions that otherwise may go unnoticed for a long time [32].

Concerns about the accuracy of sedimentation coefficients emerged again two years ago, when a ≈10% error in the elapsed time data was discovered in files produced by the manufacturer’s data acquisition software version 6.0 and associated firmware, which had been in wide-spread use for a period of nearly two years [31]. Subsequently, systematic time errors of between 0.1 to 2.0% were found in data going back more than a decade [27]. After discovery of the problem, the data acquisition software was quickly revised, but unfortunately this only restored the error to the level prior to version 6.0. Shortly after, a systematic examination of the accuracy of other data dimensions in eleven instruments revealed occurrences of temperature calibration errors by close to 3°C (which translates to a ≈7% error in *s*-values), and radial magnification errors in excess of 7% [27]. However, it was also shown that after simultaneous corrections for errors in time, radial magnification, and temperature, the range of *s*-values measured for bovine serum albumin (BSA) monomer was reduced 6-fold from 14.8% to 2.5%, and the standard deviation was improved 5-fold from 3.8% to 0.7% [27]. This is in satisfactory agreement with expectations and the demands for the technique in most current applications.

To make the required calibration measurements and corrections, the following external references were developed [27]: for the time dimension, a sufficiently accurate independent measurement is the time stamp when the data file was created, provided by the operating system of the data acquisition computer [27,31]; the rotor temperature can be logged in various configurations using miniature integrated circuits [27,33,34]; the radial magnification was measured with a precision mask installed in a cell assembly and scanned during the SV experiment [27]. To verify the mutual consistency of these calibration standards, SV experiments with a BSA sample were carried out using as a reference marker the *s*-value of the BSA monomer peak, which is baseline separated from oligomer peaks in *c*(*s*) analysis [27].

The goal of the present multi-laboratory benchmark study was to ascertain whether the observations made in the initial small calibration pilot study [27] are representative for all AUC instruments, and whether conclusions regarding the possibility of improving accuracy with external references are generally valid, widely applicable, and sufficient. For this purpose, three kits of calibration tools assembled at the National Institutes of Health were shipped to a total of 67 different laboratories, over the course of 9 months, to determine in 79 different instruments the accuracy of temperature, radial, and time calibration, and to determine the resulting distribution of *s*-values for the shared BSA reference sample. We asked whether the combination of correction factors from the external calibration references can improve the accuracy of sedimentation coefficients of BSA monomer. In addition, the comprehensive data set provided insight into several instrument performance parameters that are inaccessible from measurements in a single or a few instruments.

## Methods

### Multi-laboratory study

After an initial test phase, academic laboratories across different geographic regions were chosen as participating study sites based on the availability of an AUC (Optima XL-A or XL-I, Beckman Coulter, Indianapolis, IN, USA), interest, consideration of geographic proximity and logistics of shipping across borders, and the ability to perform the experiments in the targeted time frame of one week including receiving and shipping. Inclusion of further laboratories was stopped after approximately 9 months and 67 laboratories, balancing the statistical improvements with the benefit of a timely report of the study results, when incorporation of new data sets did not seem to significantly change the mean and standard deviation of the main calibration parameters.

Three kits were assembled each containing a DS1922L iButton temperature logger (Maxim Integrated Products, San Jose, CA, USA, purchased from Thermodata, Marblehead, MA, USA) calibrated with a reference thermometer [27]; a USB interface for the temperature logger and readout software; an aluminum holder for the iButton fitted into a standard AUC cell assembly [27]; a pre-assembled AUC cell with a precision steel mask sandwiched between quartz windows [27]; and a pre-assembled AUC cell with 400 μl of ≈0.5 mg/ml bovine serum albumin (BSA, purchased from Sigma-Aldrich, St. Louis, MO, USA, cat. No. 7030) dissolved into phosphate buffered saline (PBS; 5.60 mM Na_2_HPO_4_, 1.06 mM KH_2_PO_4_, 154 mM NaCl, pH 7.40, Corning Cellgro) in a standard 12 mm Epon double sector centerpiece with the same volume of PBS as an optically matched reference. The BSA sample was from the same stock solution for all three samples. Kits were usually shipped by express mail and courier delivery, protected against mechanical damage but not against temperature fluctuations. In addition to the kit, each laboratory was issued a detailed protocol with instructions how to carry out the following experiments:

1. An experiment to measure the temperature of the rotor spinning at 1,000 rpm: The iButton was initiated to log the temperature in 1 min intervals, installed in the aluminum holder [27] in the cell assembly, and placed into a 4-hole or 8-hole titanium rotor. This experiment was conducted with an AUC console temperature set-point of 20.0°C, for the duration of ≈3 hours, including at least 1 hour past the time when the console temperature reading equaled the set point, so as to reach thermal equilibrium of the spinning rotor in the evacuated chamber, as evidenced by the logged temperature trace. The logged temperature trace was downloaded from the logger and copied to the data analysis site.
2. A high-speed SV experiment of the radial mask and the BSA containing cell assemblies: Both AUC cells were used as received, without requiring disassembly and reassembly, inserted into a rotor and the sectors radially aligned. The SV run was started following temperature pre-equilibration of the ultracentrifuge in high-vacuum at a nominal 20.0°C for at least two hours, followed by acceleration from rest to 50,000 rpm. Signal profiles from both the radial mask and the sedimenting BSA sample cell were acquired side-by-side with the absorbance system at 280 nm and, if available, the Rayleigh interference system. The scan settings specified a time-interval of 1 min, a radial interval of 0.003 cm, a single acquisition per radius, and acquisition in intensity mode for absorbance data. (This mode was chosen because transmitted intensities are advantageous for the radial mask analysis. Prior to SV analysis of BSA, intensity data were transformed into regular absorbance data; no pseudo-absorbance analysis [35] was carried out, which is a mode not supported by the manufacturer.) Standard instrumental radial calibration through the manufacturer software was not made a part of the protocol, since it is not necessary for each run, and strictly only required after adjustments of the optical system and after replacement of a drive. In this way, the preexisting radial calibration reflects the conventional settings in each instrument.
3. Resting rotor temperature: The possibility for temperature reference experiments with the resting rotor was discovered and published after the present study was initiated [34]. In order to validate this approach on a larger scale, a subset of the remaining laboratories carried out additional temperature experiments on the resting rotor following the procedure described in detail in [34]. Briefly, the iButton was placed on the counterbalance of the 8-hole rotor resting in the evacuated rotor chamber, rotated such that the counterbalance was located above the radiometer, temperature equilibrating without the monochromator arm at a nominal AUC console set temperature of 20.0°C [34].

In all, 129 complete data sets with experiments (1) and (2) were acquired from a total of 79 instruments in 67 laboratories in 15 countries who received the kit; in 9 cases only partial information could be retrieved; in 3 sites the experiments failed to produce sedimentation boundary data and were not included. 15 sites carried out experiments (3) in addition to (1) and (2).

For a test of consistency of the kits, experiments (1) and (2) were run with each kit at the time of assembly in the originating laboratory at the National Institutes of Health, Bethesda, in the same instrument. In the middle of the study, all kits were shipped back and experiments were carried out simultaneously with all kits side-by-side in the run. This consistency test was repeated at the end of the study.

(We note that equipment, instruments or materials are identified in this paper in order to adequately specify the experimental details; such identification does not imply recommendation by author institutions, nor does it imply the materials are necessarily the best available for the purpose.)

### Data analysis

In order to exclude variation from data analysis, all data analysis was carried out by a single operator, using the same method.

From the temperature logs in experiment (1) it was possible to confirm that the temperature had reached a steady-state while the rotor was spinning at 1,000 rpm in the evacuated AUC rotor chamber set at 20.0°C. Occasionally an oscillation within 1 bit (corresponding to 0.06°C) was observed, in which case the reading was averaged. Previously determined iButton calibration constants based on a NIST reference thermometer were applied to the measured values [27]. The observed deviation of the rotor temperature from the nominal 20.0°C was used to calculate temperature correction factors based on the temperature dependence of the viscosity of water. The temperature correction factors were applied as multiplicative factors to the observed *s*-values [27], and so corrected values are labeled with index ‘20T’.

The radial magnification correction factor was determined from an analysis of the radial mask scans collected in experiment (2) at 50,000 rpm, as described previously [27]. The software MARC was used to recognize the 14 equidistant edges of the mask in the scans, and to plot and analyze the distance between experimental edge radii against the known true distance of the precision mask. From the slope of this plot the calibration correction factor for radial magnification was determined. This radial magnification correction factor was used as a multiplicative correction to the apparent *s*-values [27], indicated with index ‘r’.

When plots of apparent *vs* true edge distances were not well described with a linear fit, the smallest satisfactory polynomial (usually quadratic, not more than cubic) was fitted to the edge displacement data. The resulting parameters were used to define a non-linear back-transformation, which was applied directly to the radial positions of all scan file data points. The corrected scan files were saved and used for further data analysis.

The scan time correction factor was determined by comparison of the differences between scan header data and the intervals between the time-stamps of scan file creation [31]. Time corrected scan files were generated using SEDFIT or REDATE, and results from the analysis of time corrected data were labeled with index ‘t’. Scan velocity errors, arising from the finite duration of a single scan in the absorbance system, were taken to be constant 0.18%, as determined previously for the scan settings specified for the sedimentation experiment (2) [27,36]. These are absent for the interference system. Corrected values are indicated with a subscript ‘v’.

Scan files for the BSA experiment were analyzed with the *c*(*s*) method [37]. We have excluded questions of alternative data analysis strategies in order to focus on parameters related to instrument performance. As in previous calibration studies [27,31], scans representing the entire sedimentation process were loaded in SEDFIT, and both the meniscus and the signal-average frictional ratio were refined in the fit, allowing for TI noise in absorbance data and TI and RI noise in interference data. *s*-values of the BSA monomer peak were determined by integration of the baseline-separated *c*(*s*) peak in SEDFIT, and multiplied by the appropriate correction factors. Uncorrected values are labeled ‘raw’, and values after joint correction for temperature, radial magnification, scan time and velocity are labeled ‘20T,t,r,v’. No further solvent correction to conditions of water was applied. A table with the data can be found in the **S1 Table**.

Software used in the present study are SEDFIT (sedfitsedphat.nibib.nih.gov) for the SV data analysis; MARC (Mask Analysis for Radial Calibration, by Dr. Huaying Zhao, manuscript in preparation) for the analysis of calibration mask scans [27]; SEDFIT or REDATE (by Dr. Chad Brautigam, http://biophysics.swmed.edu/MBR/software.html) for the time-stamp corrections [31]; SEDNTERP for the calculation of solvent viscosity (by Dr. John Philo) [38]; and GUSSI (by Dr. Chad Brautigam, http://biophysics.swmed.edu/MBR/software.html, manuscript in preparation) for plotting SV results.

## Results and Discussion

The present study generated much data on different aspects of accuracy and precision of ultracentrifugal measurements. As it is a prerequisite for all further analysis, we will first present evidence supporting the stability with time of the common sample and artifacts that were shared between laboratories. This is followed by the analysis of accuracy of data acquisition in the different data dimensions: time, radius, and temperature. Separate from these calibration measurements, the sedimentation experiment of BSA offers independent insight into instrument performance, and the results are shown with regard to the overall quality of fit to the SV data and interesting isolated cases. The key of the present study is the test whether the corrections for calibration errors of the different data dimensions can successfully explain and reduce the spread of observed sedimentation coefficients of BSA monomer. The combination of corrected *s*-values and experimental observation of the meniscus position then offers an opportunity to verify whether the radial corrections for radial magnification, neglecting translation errors, are sufficient. Finally, we will discuss other attributes of the family of experimental SV data that are not directly related to the calibration corrections, but reflect on the instrument-to-instrument variation of other quantities, including the signal magnitudes in absorbance and interference, the determination of the BSA dimer fraction, and apparent molar mass and boundary spread.

### Integrity of the calibration kits

**Fig 1:**
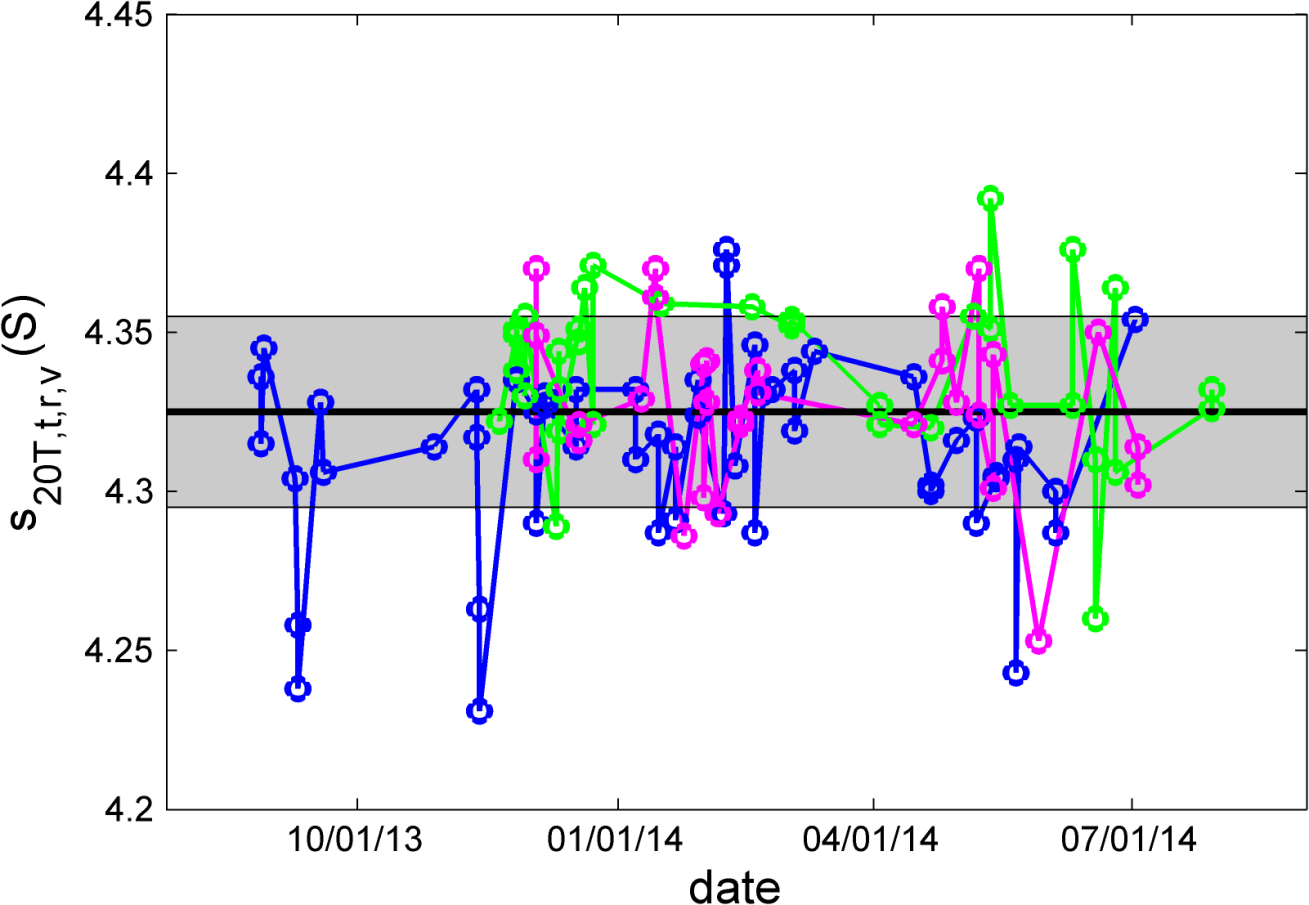
Absence of a long-term trend in *s*_*20T*,*t*,*r*,*v*_-values of the BSA monomer with time of experiment for the three kits (blue, green, and magenta). Highlighted as bold solid line is the overall average, and the grey area indicates one standard deviation.

A key assumption of the present study was that the kits are stable with time, for example, that the BSA solution did not degrade from exposure to high and low temperature during shipping, that the cell assemblies with the BSA sample and with the radial mask remained mechanically intact, and that the performance of the temperature loggers did not deteriorate with time due to declining battery power. To verify the absence of such confounding factors, we examined the trend curves of measured parameters with time. For example, **Fig 1**shows the *s*_*20T*,*t*,*r*,*v*_-values for the BSA monomer after calibration corrections as a function of experiment time. No trend could be observed for any of the kits. Similarly, no trend could be recognized with temperature measurements and radial calibration factors (**S1 Fig** and **S2 Fig)**. Based on the trend of the best-fit meniscus positions, in one kit a small loss of sample at a rate of <5 μl/year may possibly have occurred, but none is evident in the others (**S3 Fig**). Further, except trivially for the meniscus position, none of these factors offered any indication for a kit-dependent bias.

BSA is well-known to form irreversible oligomers. However, the *c*(*s*) analysis can resolve monomer and oligomeric species into baseline-separated peaks, and the measured BSA monomer *s*-value would therefore not be affected by such slow irreversible aggregation. (Similarly, small fragments from protein degradation can typically be baseline resolved.) Nevertheless, we examined the resulting monomer signal intensity and the fraction of dimer (where the dimer is clearly resolved from monomer and higher order aggregates) as a function of time. No correlation was found for the monomer signal (**S4 Fig**), however, a slight trend may be discerned in the dimer fraction possibly decreasing by about 2% per year (**S5 Fig**). These results demonstrate that the sample was sufficiently stable in PBS for the duration of the multi-laboratory study.

In the middle of the multi-laboratory study, and again at the end of the study, the three kits were run side-by-side in the same rotor in the same instrument. In experiment (1), the iButtons readings showed maximal differences of 0.07°C relative to each other both in the middle and at the end of the study, respectively, each iButton producing the identical reading at the two time points. In experiment (2), scan time corrections were essentially identical, and radial correction factors coincided within < 0.15% and < 0.24% at the midpoint and end of the study, respectively. Final corrected *s*_*20T*,*t*,*r*,*v*_-values from each kit’s BSA cell assembly were within 0.39% and 0.37% of each other at the midpoint and end of the study, respectively. This provides further evidence for the integrity of the calibration kits with time and the consistency among the kits.

### Time

The distribution of errors in the elapsed time since start of the centrifuge run reported in the scan file headers was bimodal. For most of the data sets these were small, but not negligible ranging from 0.12% to 0.3% with a mean and standard deviation of 0.16% ± 0.05%. This is consistent with the previously reported errors in the updated data acquisition software version for the scan settings used in the present study. However, 9 out of 79 instruments exhibited a much larger scan time error of 10.34% ± 0.43%, suggesting the operation of the previous, faulty data acquisition software/firmware version. Two instruments operated with user interface software versions 3.x (originally distributed in the mid 1990s) produced data with elapsed time values in error by a factor 10 (i.e. an error of 1000%), yet seemingly correct ω^2^t values. Uncorrected *s*-values for these instruments were not included in the study so as not to contaminate the statistics with these easily recognizable outliers.

### Radial magnification

**Fig 2:**
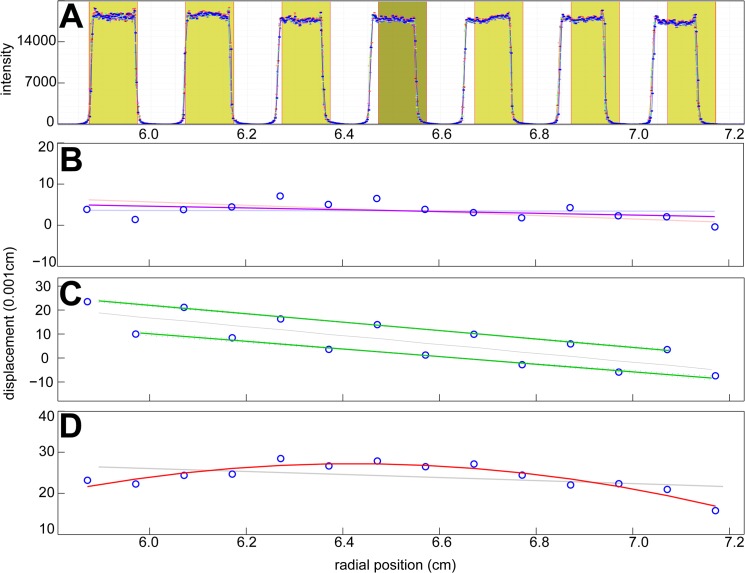
Examples for the determination of radial magnification errors. (A) Radial intensity profile measured in scans of the precision mask. Blue lines are experimental scans, and shaded areas indicate the regions expected to be illuminated on the basis of the known mask geometry. In this example, the increasing difference between the edges corresponds to a calculated radial magnification error of -3.1%. (B—D) Examples for differences between the experimentally measured positions of the light/dark transitions (blue circles, arbitrarily aligned for absolute mask position) and the known edge distances of the mask. The solid lines indicate the linear or polynomial fit. (B) Approximately linear magnification error with a slope corresponding to an error of -0.04%. Also indicated as thin lines are the confidence intervals of the linear regression. (C) A bimodal shift pattern of left and right edges, likely resulting from out-of-focus location of the mask, with radial magnification error of -1.7%. (D) A non-linear distortion leading to a radial magnification error of -0.53% in the *s*-values from the analysis of back-transformed data. The thin grey lines in C and D indicate the best linear fit through all data points.

Interference and absorbance radial magnification correction factors were determined from scans of the precision mask as previously described [27]. Representative intensity scans are shown in **Fig 2A**, and **Fig 2B–2D**show examples for the displacement of the measured edge position of the mask. Most instruments showed a small, linearly increasing offset relative to the known edge distances, from which the magnification correction factor could be determined via the slope of the best-fit straight line (e.g., **Fig 2B**).

In some instruments the left and right edges exhibited a constant offset relative to each other (**Fig 2C**) suggesting out-of-focus location of the mask in the cell assembly. In these cases all left edges and all right edges were analyzed separately, and the magnification factor was taken as the average between the slopes of these plots. This was only observed in interference data.

A minority of instruments (18%) exhibited moderate non-linear distortions in the absorbance data, such as shown in **Fig 2D**. In these cases the radial scan files were radially back-transformed on the basis of a quadratic or cubic fit to the mask edge locations. The resulting *c*(*s*) analysis of the back-transformed data usually exhibited a slightly improved root-mean-square-deviation (rmsd) as compared to the analysis of the raw data. Solely for the quantitative comparison, the ratio of apparent *s*-value from the analysis of the radially uncorrected and the radially back-transformed data was taken as an average magnification correction factor.

**Fig 3:**
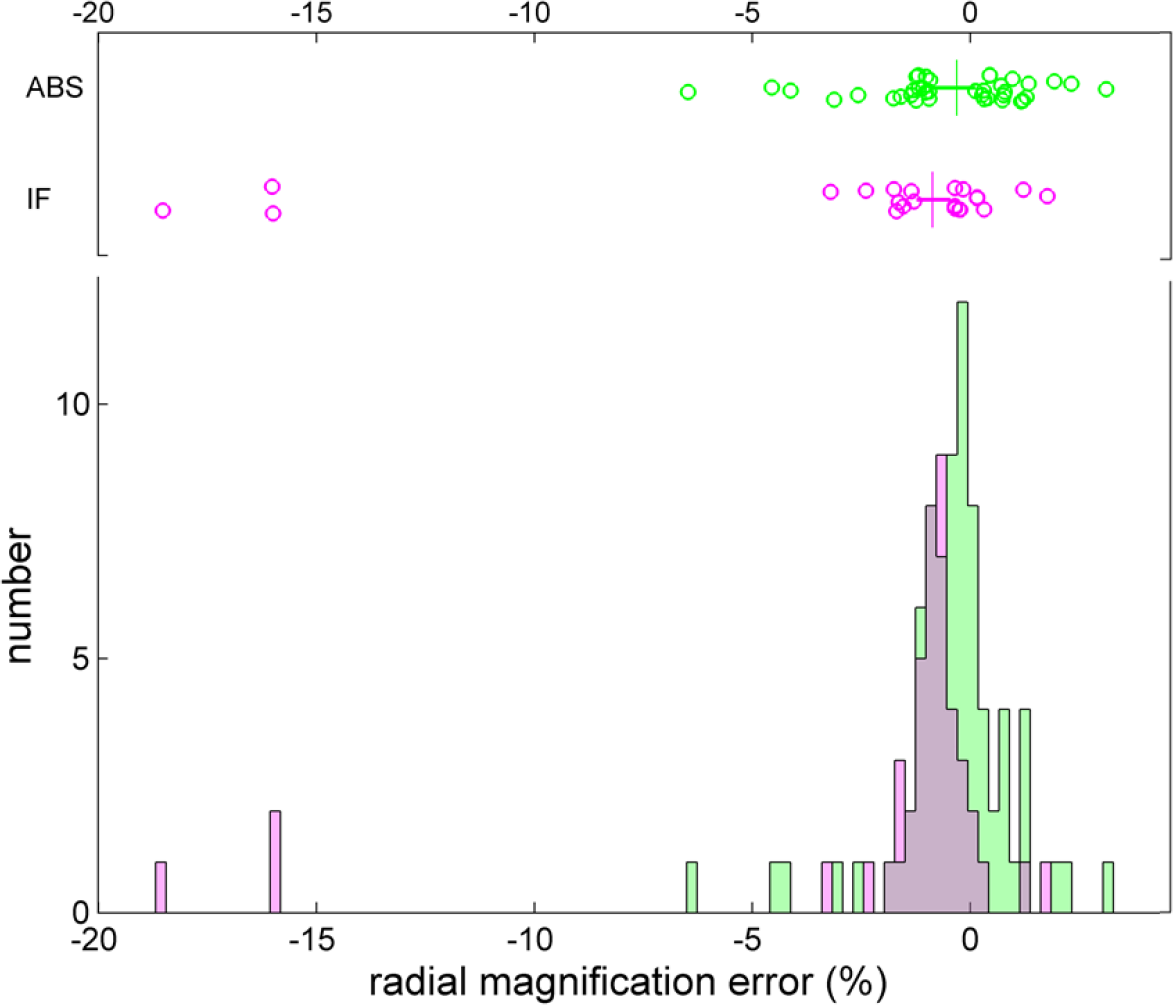
Magnitude of the radial magnification correction obtained with the absorbance system (green) and the interference system (magenta). The box-and-whisker plot above the histogram indicates the central 50% of the data as solid horizontal line and draws data in the smallest and highest 25% percentiles as individual circles. The median is displayed as vertical line. The mean and standard deviations are -0.43% ±1.36% for the absorbance system, and -0.75% ± 0.82% for the interference system (once the three outliers are excluded).

The distribution of radial correction factors is shown in **Fig 3**. Overall, it shows a small bias toward underestimation of radial distances, requiring a positive mean correction of 0.95%, but with a large scatter and a standard deviation of 2.77%. It is of interest to consider errors in the absorbance system and the interference system separately. Calibration errors in the absorbance system ranged from -6.5% to +3.1%, with a mean of -0.43% and a standard deviation of 1.36%. In the interference system, a mean magnification correction factor of 1.85% with standard deviation of 4.14% was determined. Interestingly, the large spread of values was dominated by three instruments exhibiting a very large error of 16–19%, which, incidentally, exceeds the error that could be caused by an operator misidentifying the relevant counterbalance edges during IF calibration. Possible contributing factors to this error include rotation of the counterbalance and/or its sectorial masks. If the outliers are excluded from the statistics, the remaining magnification corrections for the interference system had a mean of -0.75% and a standard deviation of 0.82%. The significantly lower variation in the interference optics as compared to the absorbance optics likely reflects the higher optical resolution in the former and the lack of moving parts.

**Fig 4:**
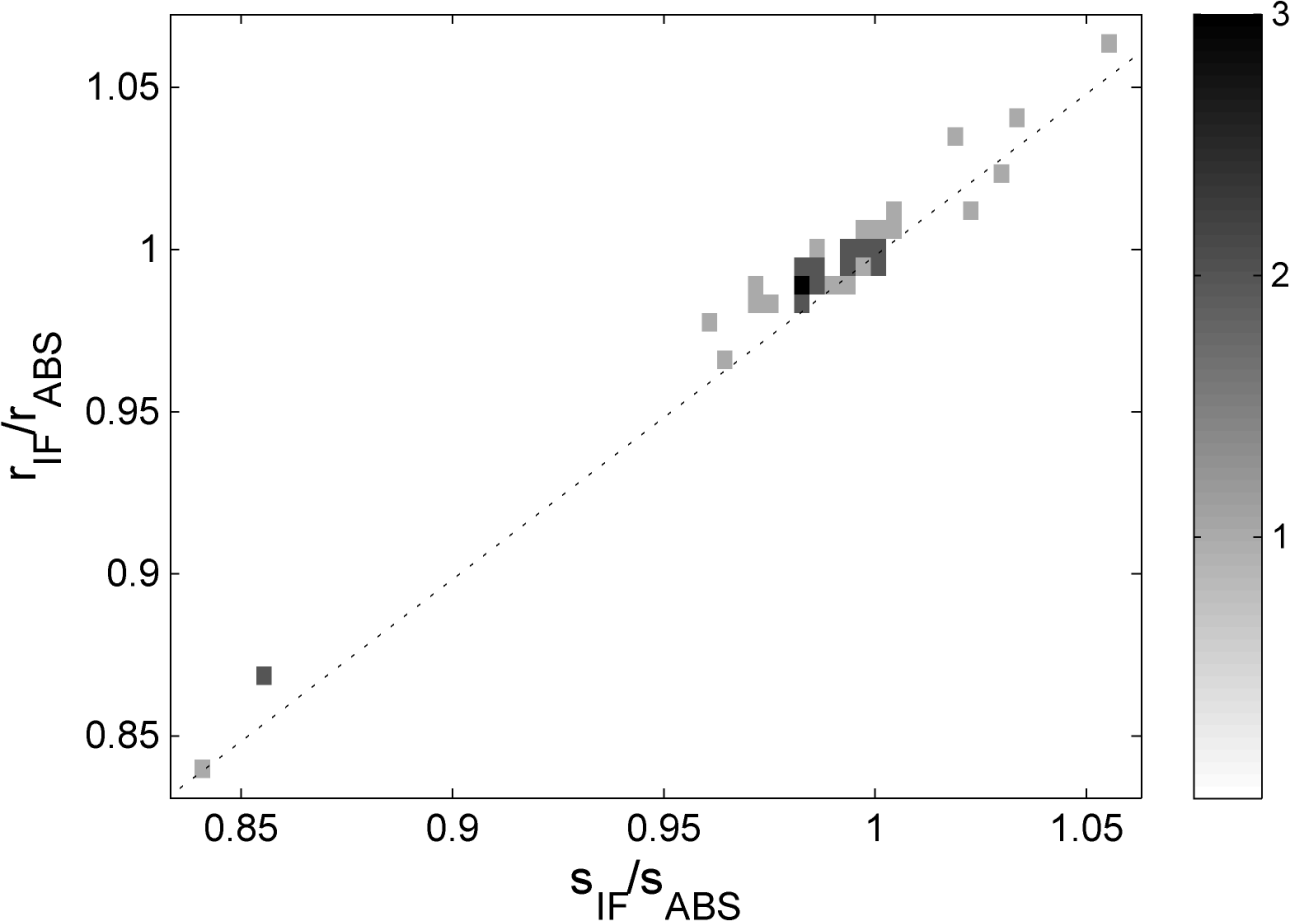
Correlation between the ratio of uncorrected *s*-values for the BSA monomer peak from the simultaneously acquired interference and the absorbance data (*s*_IF_/*s*_ABS_) and the ratio of corresponding radial magnification correction factors measured independently with the steel mask (r_IF_/r_ABS_). Squares are a histogram with frequency as indicated in the sidebar. The dotted line indicates the ideally expected relationship assuming perfect measurements of *s*-values and radial magnification correction factors.

Radial magnification errors will affect directly and proportionally the measurement of sedimentation velocity, and in those instruments where data from both optical systems are available, the radial magnification errors will be different for the different optical systems. By contrast, the uncorrected *s*-values from the BSA monomer peak in *c*(*s*) analysis, measured either in the absorbance system or in the interference system, are subject to the same errors in scan time, solvent viscosity (temperature), and the same effects of imperfect alignment- or temperature-driven convection. Thus, one would expect that the ratio of the uncorrected *s*-values, s_IF_/s_ABS_, to be proportional to the ratio of radial magnification correction factors, r_r,IF_/r _r,ABS_. A plot of both quantities, for 42 instruments for which this data is available, is shown in **Fig 4**. The strong correlation independently validates the veracity of the radial magnification correction factors determined from the analysis of the mask scans.

### Temperature

**Fig 5:**
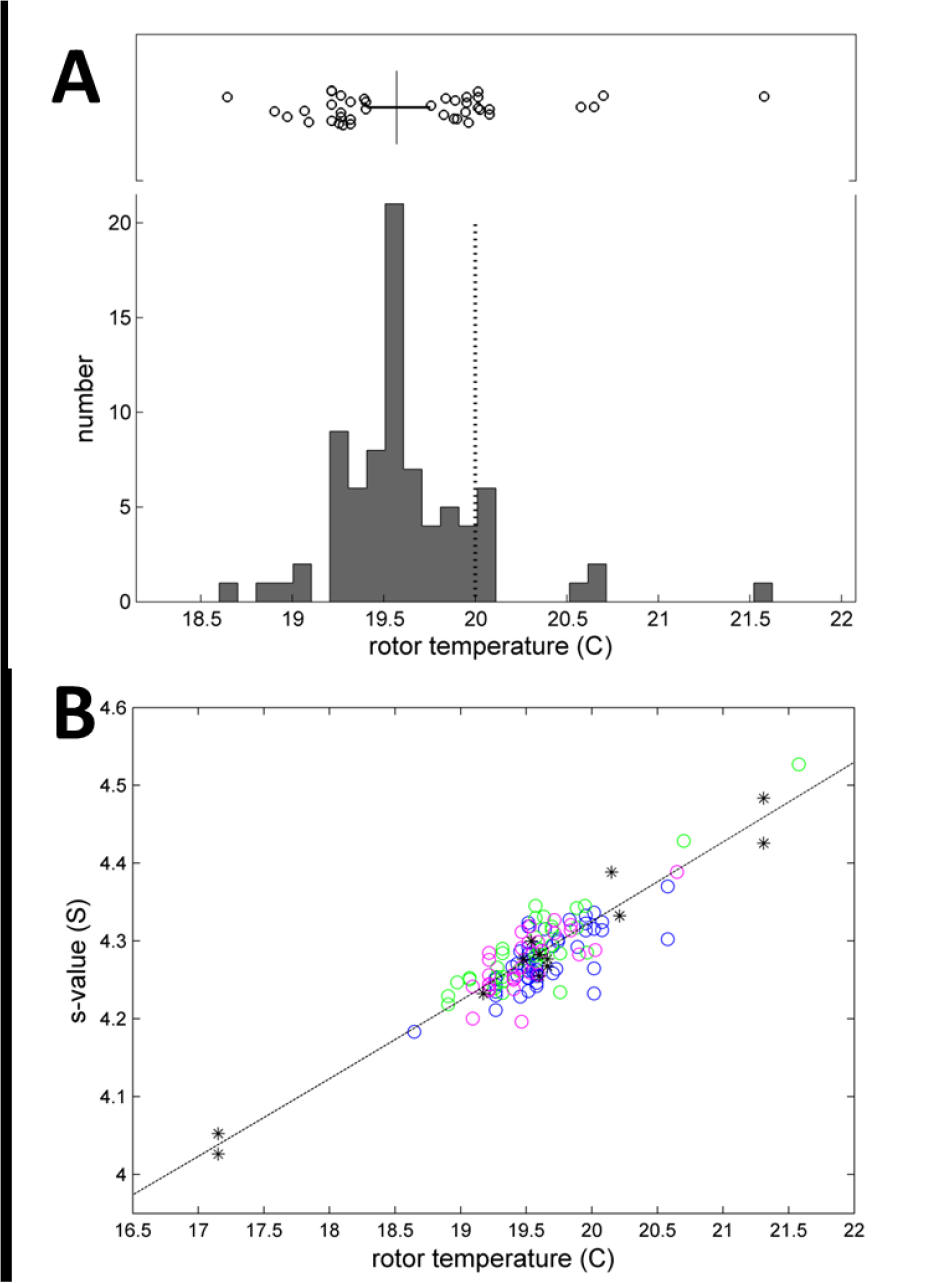
Analysis of the rotor temperature. (A) Temperature values obtained in different instruments of the spinning rotor, as measured in the iButton at 1,000 rpm after temperature equilibration, while the set point for the console temperature is 20°C (indicated as dotted vertical line). The box-and-whisker plot indicates the central 50% of the data as solid line, with the median displayed as vertical line, and individual circles for data in the upper and lower 25% percentiles. The mean and standard deviation is 19.62°C ± 0.41°C. (B) Correlation between iButton temperature and measured BSA monomer *s*-values corrected for radial magnification, scan time, scan velocity, but not viscosity (symbols). In addition to the data from the present study as shown in (A) (circles), also shown are measurements from the pilot study [27] where the same experiments were carried out on instruments not included in the present study (stars). The dotted line describes the theoretically expected temperature-dependence considering solvent viscosity.

The temperature set point was adjusted at the AUC console to a nominal 20.0°C in all experiments. We have previously established the precision of iButton readings of the rotor temperature to be 0.06°C or better, and estimated the accuracy to be 0.1°C—0.2°C [27,33]. In the present study, the iButton reading logged at steady-state after temperature equilibration at 1,000 rpm ranged between 18.64°C and 21.58°C, with a mean of 19.62°C and a standard deviation of 0.41°C (**Fig 5A**). Although the average is within the instrument specifications for temperature accuracy of ± 0.5°C, and the standard deviation is less than 0.5°C, most instruments (49 out of 79, or 62%) exhibited temperature deviations outside the range from 19.5 to 20.5°C. With time, calibrations can diverge from original specifications. The temperature errors led to an average viscosity correction factor of 0.9770, i.e. a temperature error contribution of 2.30% ± 0.95% to the *s*-values. This is independently confirmed by the clear correlation between the iButton temperature and the measured *s*-values of BSA monomer after all corrections except for temperature (**Fig 5B**, in which we have included instruments from the pilot study which exhibited more extreme temperature values).

In 15 sites, additional temperature measurements were carried out on the resting rotor, following a more recent protocol [34]. The obvious advantage of this configuration is that no custom fabricated iButton holder is required to fit them into the standard barrel [27], and no rotor handle modification is required [33], such that commercial temperature loggers can be directly applied to measure the temperature calibration offset of the AUC. On average, the absolute difference between the spinning and resting rotor was 0.12°C, with a maximal deviation in one instrument of 0.31°C. This confirms that a reasonably accurate measurement of the rotor temperature is possible in the resting rotor.

**Fig 6:**
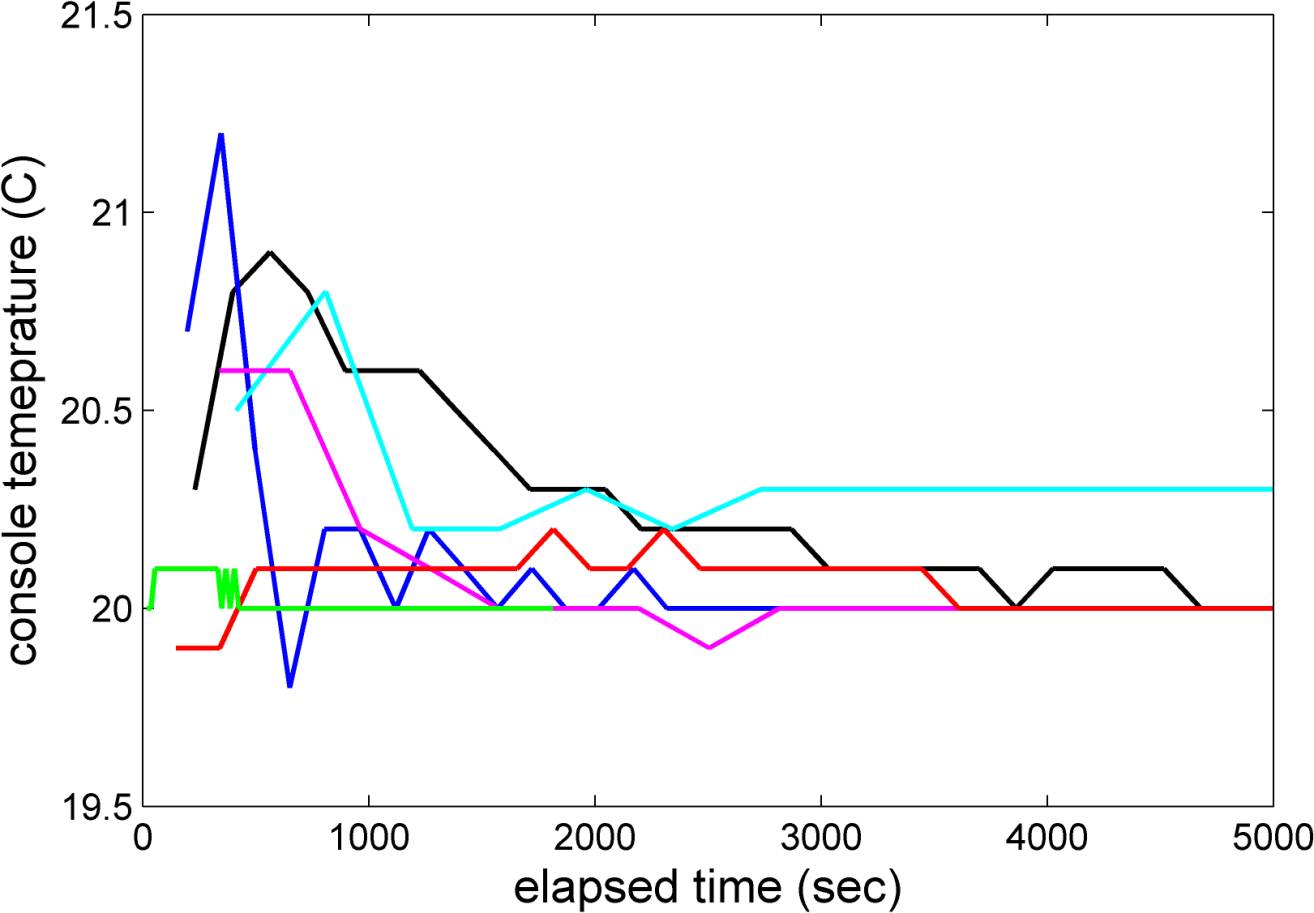
Examples of transient changes in the console temperature reading during the SV experiment, as saved in the scan file data. For comparison, the maximum adiabatic cooling of -0.3°C would be expected after approximately 300 sec, recovering to the equilibrium temperature after approximately 1,200 s (see Fig 3 in [33]).

In most instruments the scan files acquired during the high-speed sedimentation experiment (see Methods Experiment #2) revealed an initial transient positive jump in the console temperature, most commonly in the 0.1–0.5°C range, but up to 0.8°C, before the temperature slowly decreased toward the set-point of nominal 20.0°C (e.g., magenta and blue trace in **Fig 6**). (An extreme case with a jump by 1.4°C occurred in one instance, which was rejected and the experiment repeated.) This is opposite to the expected transient negative temperature jump from adiabatic stretching and cooling of the rotor upon rotor acceleration [33,39]. In several instances, the experiment was repeated with even more exhaustive temperature equilibration prior to the run, usually without significant improvement. This mimics observations made in instruments at the National Institutes of Health, where such behavior was occasionally observed at the beginning of an SV run despite exhaustive temperature equilibration, which fortuitously happened in a few cases when the rotor handle iButton was engaged and logging rotor temperature data independently. In one case, during the transient temperature increase, the iButton temperature log confirmed the console temperature drift, but in another case the transient increase in the AUC temperature reading was not confirmed (unpublished data). In view of the latter case, it is possible that these transient temperature jumps are caused by radiometer artifacts due to the presence of poor vacuum [39], for example, during initial outgassing of the diffusion pump oil, and therefore may not reflect the true rotor temperature. Real transient temperature drifts in the beginning of the experiments would be of concern for the accuracy of the measured sedimentation coefficients due to the potential of convection. False readings would raise the secondary concern of excess cooling of the rotor.

In order to examine whether these transient temperature drifts are real and influence the results of a sedimentation velocity experiment, we have plotted the final corrected *s*_*20T*,*t*,*r*,*v*_-values of the BSA monomer peak versus the magnitude of the initial temperature jump for instruments that showed a positive temperature jump (Panel A in **S6 Fig**), but no obvious correlation could be discerned. Convection is sometimes visible in characteristic deviations of the data from the best-fit in the boundary region of early scans. To exploit this feature as a marker for the presence of convection, residuals of the fit in the 1 mm of the solution column at lowest radii adjacent to the fitting limit were compared with the overall residuals. However, again, no correlation with the magnitude of the apparent initial temperature jump was observed (Panel B in **S6 Fig**). This supports the notion that the initial temperature drifts may not be real in many cases, or may be too small or transient to be of significant consequence for the analysis.

In a few instruments, after the initial transient increase, the temperature reading did not return to the set-point of 20.0°C (e.g., cyan trace in **Fig 6**). In these cases, the difference between the temperature set point of 20.0°C to the final run temperature recorded in the scan files was considered real, and used to apply additional small viscosity corrections to the *s*-values.

### Quality of fit of the BSA sedimentation velocity experiment

**Fig 7:**
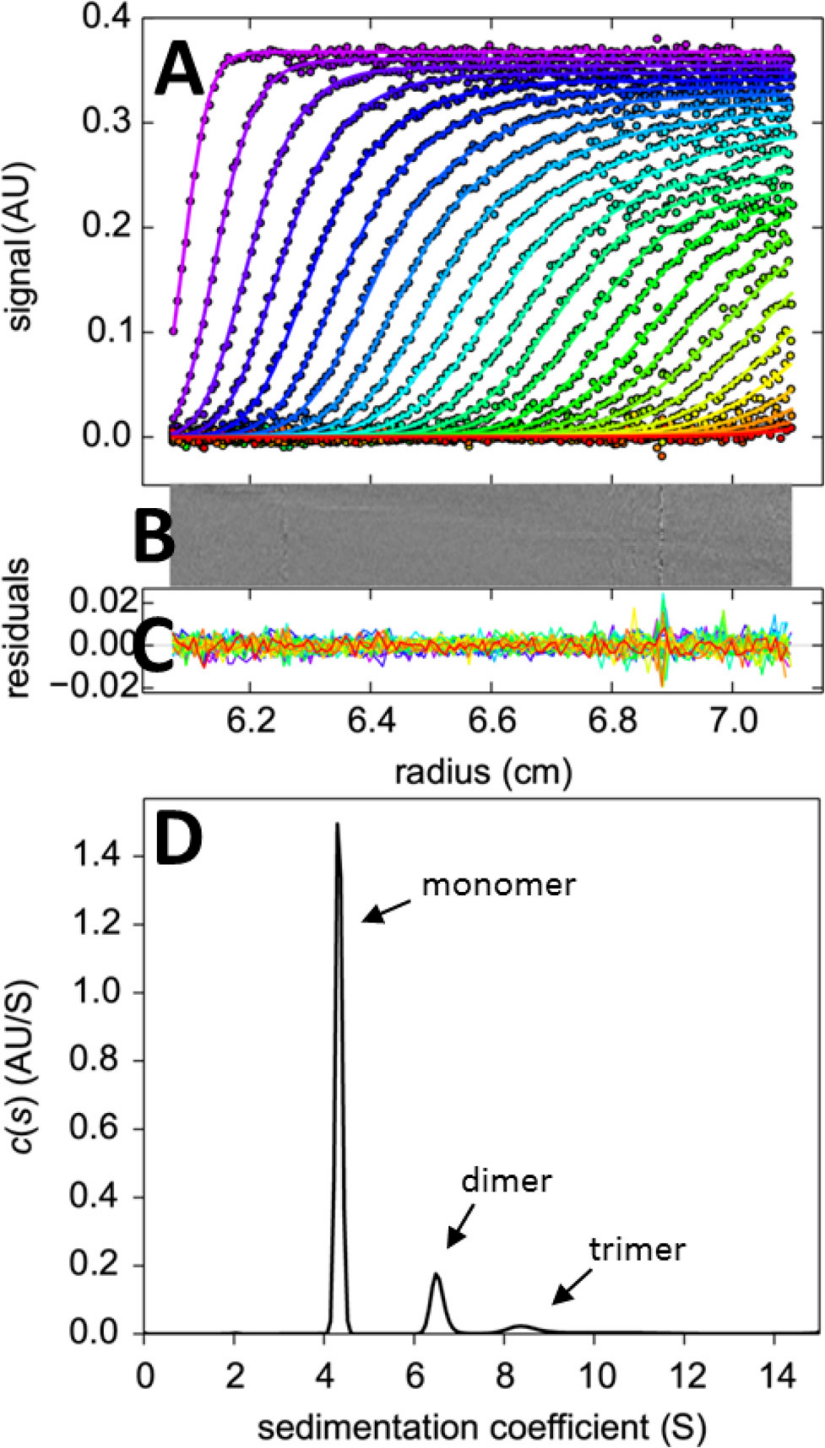
Example for the analysis of absorbance data from the sedimentation velocity experiment of BSA. (A) Absorbance scans (symbols) and best-fit *c*(*s*) model at different points in time indicated by color temperature. (B and C) Bitmap and overlay of the residuals of the fit. (D) *c*(*s*) sedimentation coefficient distribution showing peaks for monomer, dimer, trimer, and traces of higher oligomers.

**Fig 8:**
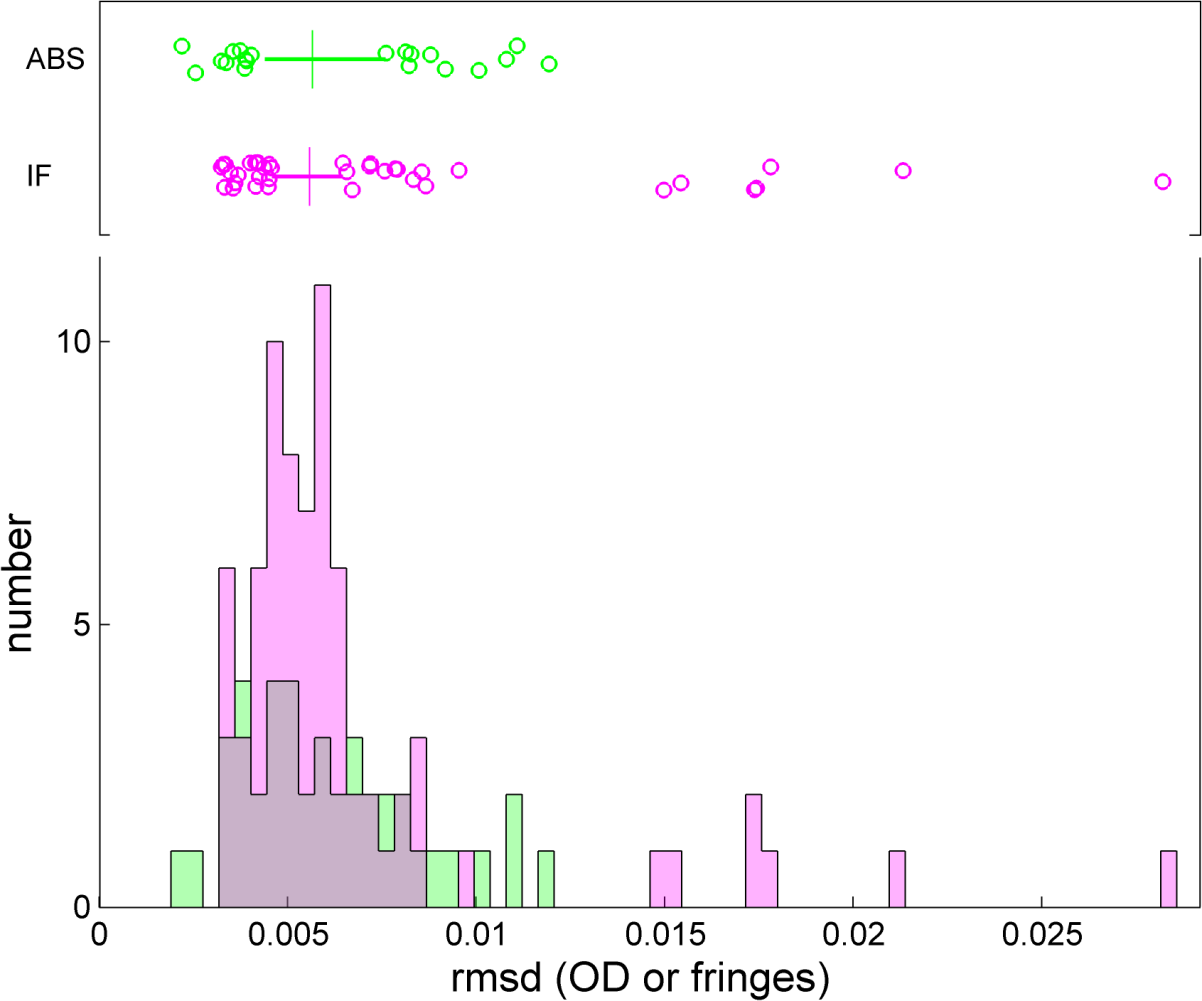
Root-mean-square deviation of the best-fit *c*(*s*) model of the BSA sedimentation experiment when scanned with the absorbance system (green) and the interference system (magenta). The box-and-whisker plot indicates the central 50% of the data as solid line and draws the smaller and larger 25% percentiles as individual circles. The median is displayed as a vertical line.

The design of the study was such that the analysis of the sedimentation boundaries recorded from the BSA sample would allow for an assessment of the completeness and internal consistency of the external calibration corrections in time, temperature, and radial magnification. Generally the quality of fit of the experimental SV data was excellent (for a representative BSA sedimentation velocity absorbance analysis, see **Fig 7**), with an average rmsd of 0.0067 OD in the absorbance system and 0.0061 fringes in the interference system. However, as shown in **Fig 8**, the distribution of rmsd values showed a minority of outliers. Some of the outliers in the absorbance data set can be traced simply to very noisy data resulting from low signal intensities below 800 counts. However, at 800 counts and above, no correlation between the rmsd of the fit and reported signal intensity counts was found (data not shown). In other cases, the rmsd was particularly high in the initial scans close to the meniscus region, pointing to convection as an origin of the misfit (e.g., **S7 Fig**). Interestingly, in a few instruments, the interference data exhibited an unexplained pattern of sloping plateaus (e.g., **S8 Fig**), which required truncation of the radial range used for analysis and correlated with increased errors in the determination of boundary magnitudes (data not shown). Finally, a few interference data sets exhibited wavy patterns superimposed onto the boundary shapes, but without causing excessively high rmsd or outlier *s*- and *M*-values (data not shown). Overall, in 11 out of 119 experiments the data acquisition was either not attempted, failed, or produced data sets where the *c*(*s*) model did not lead to baseline separation between the BSA monomer and dimer peak; these data sets were not included in the analysis of *s*-values.

### Consistency of calibration and BSA monomer sedimentation coefficients

**Fig 9:**
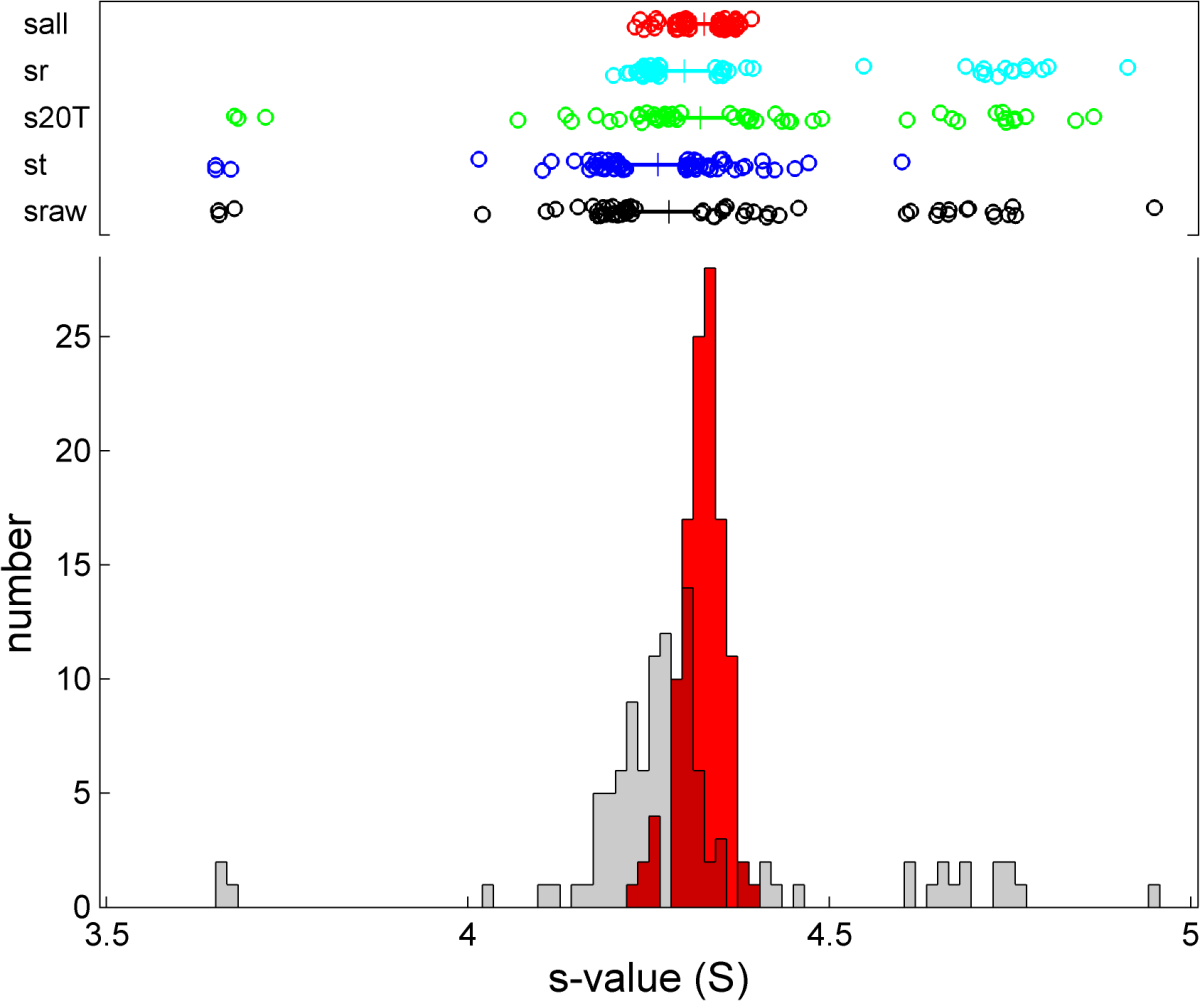
Histogram and box-and-whisker plot of *s*-values of the BSA monomer after different corrections: Raw experimental *s*-values (black, with grey histogram), scan time corrected *s*_*t*_-values (blue), rotor temperature corrected *s*_*20T*_-values (green), or radial magnification corrected *s*_*r*_-values (cyan), and fully corrected *s*_*20T*,*t*,*r*,*v*_-values (red with red histogram). The box-and-whisker plots indicate the central 50% of the data as solid line and draw the smaller and larger 25% percentiles as individual circles. The median for each group is displayed as a vertical line.

The black box-and-whisker plot and grey histogram of **Fig 9**show the distribution of *s*-values prior to any calibration correction. Values ranged from 3.655 S to 4.949 S, with a mean and standard deviation of 4.304 ± 0.188 S (4.4%). The blue, green, and cyan box-and-whisker plots show the distributions when data were corrected only for scan time errors, temperature errors, or radial calibration errors, respectively. While the individual corrections did not significantly improve the consistency of the *s*-values, the joint correction for all three factors, additionally including the small correction for scan velocity in the absorbance data, provided a substantial improvement, as shown in the red box-and-whisker plot and histogram. The range of *s*_*20T*,*t*,*r*,*v*_-values is reduced, compared to the raw data, by a factor ≈7, and the standard deviation reduced by a factor ≈6, arriving at a mean of 4.325 S ± 0.030 S (0.7%) for the BSA monomer at 20°C in PBS. This clearly supports the usefulness and improvements in accuracy from the external calibrations. Furthermore, the absence of remaining outliers suggests that all major sources of systematic errors are accounted for.

### Absolute radial position

**Fig 10:**
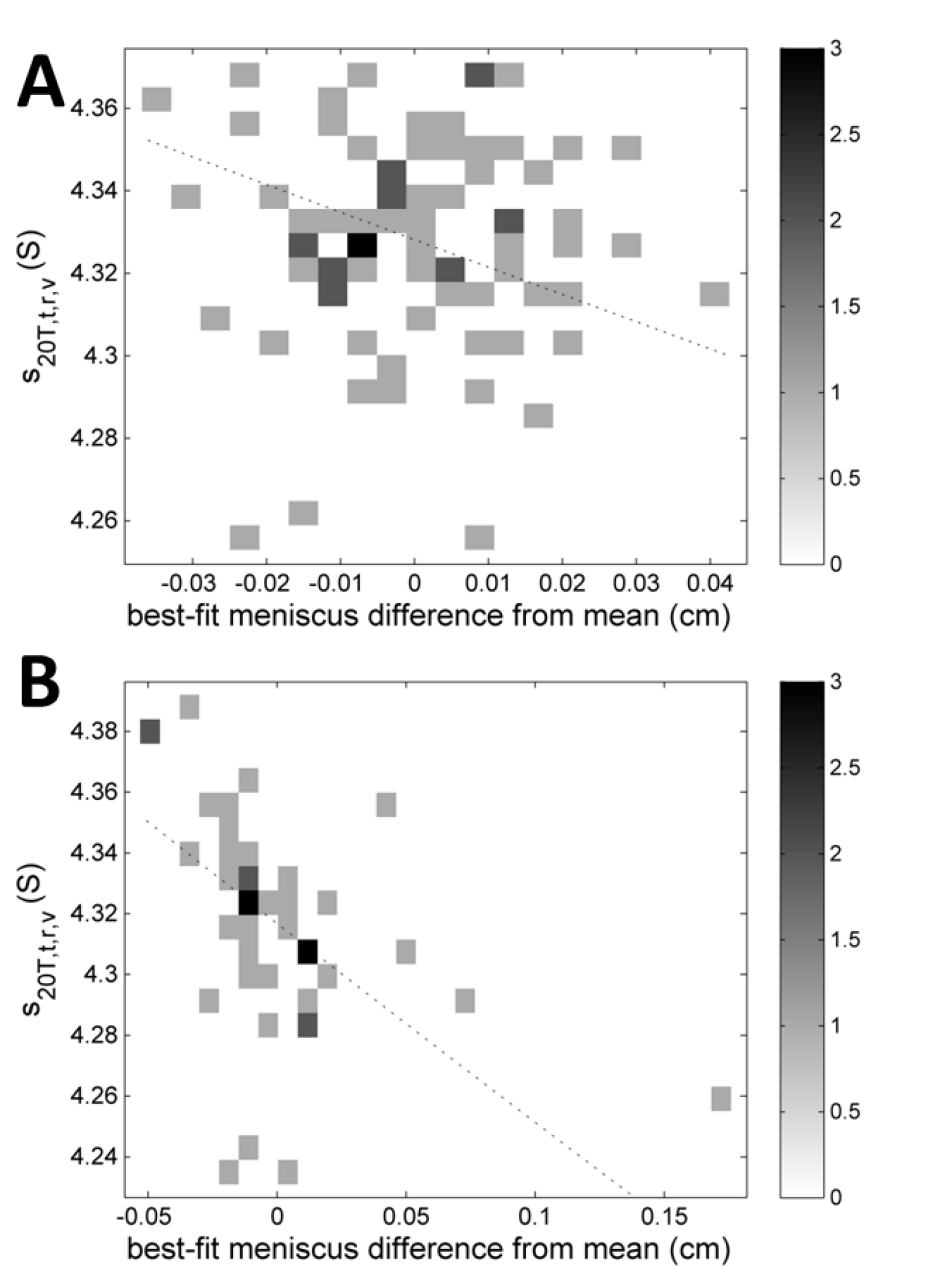
Correlations of the *s*_*20T*,*t*,*r*,*v*_-values of the BSA monomer with the difference of the best-fit meniscus from the mean meniscus value, separately for absorbance data sets (A) and interference data sets (B). The difference of the best-fit meniscus to the mean was calculated separately for each kit, to eliminate offsets due to different sample volumes in each kit, and then merged into groups for the optical systems. Data are shown as a histogram with frequency values indicated in the colorbar. The dotted lines show the theoretically expected dependence of the apparent *s*-value on errors in the absolute radial position.

Independent of the radial magnification, radius values can be subject to a translation error, i.e. a constant displacement uniformly offsetting all recorded radii. Even though the radial mask could in principle provide information of instrument-to-instrument variations in the radial translation, another marker is the meniscus position of the BSA sample as determined implicitly by the boundary movement through its best-fit position in the *c*(*s*) analysis. It allows for an excellent measurement of the absolute radial position because of the absence of some optical edge effects, and is very well determined implicitly from the many scans reporting on the boundary movement. From the analysis of a single SV data set, the statistical error of the best-fit meniscus position (propagated from noise in the acquired concentration signals) is typically less than 0.002 cm, incidentally a value less than the average distance between data points in absorbance scans. It is obvious that the meniscus position for the different kits will vary slightly due to pipetting errors in the sample volumes, but the true value is identical for the measurements of the same kit. When calculated as the standard deviation of the difference between the calculated meniscus positions from the mean value of its kit, an estimate for the precision of the meniscus position of 0.015 cm in the absorbance system is obtained. For the interference system, the corresponding value is larger at 0.037 cm (**Fig 10**). Since these values are much larger than the precision of the estimates from a single data set, we believe they reflect on the radial calibration in the form of radial translation errors.

The translation error propagates into errors in the gravitational force calculated on the basis of the distance from the center of rotation, and therefore should impact AUC data analysis much less than magnification errors, which impact the recorded distances traveled. For example, data that are erroneously attributed to higher radii will be assumed to be at higher centrifugal fields, leading to an underestimate of the *s*-value (as it is defined as the velocity normalized by the gravitational field) (dotted lines in **Fig 10**). Given the precision in the meniscus values of 0.015 cm and 0.037 cm, respectively, the corresponding errors in *s*-values would be 0.23% or 0.57%, which is less than the standard deviation of *s*_*20T*,*t*,*r*,*v*_-values of the BSA monomer. A plot of the experimental *s*_*20T*,*t*,*r*,*v*_-values *versus* meniscus deviations from kit-average is not inconsistent with a correlation, but from the correlation plot of the experimental data in **Fig 10**we can conclude that errors in the absolute radial position appear to be of minor influence on the spread of *s*_*20T*,*t*,*r*,*v*_-values.

### Photometric precision and fringe shift variation

One additional data dimension for which accuracy is often taken for granted is the signal magnitude, i.e. the absorbance and fringe displacement values for a given concentration. The manufacturer does usually not provide specifications for photometric accuracy; though two of the instruments in the present study were subject to the separately offered photometric certification.

Since we did not include the measurement of a photometric standard, the present study offers insight in these parameters not in terms of absolute accuracy. However, we can study their precision, from the statistics of the signals generated by the common BSA sample. The data from the different kits could vary slightly as each was prepared in separate dilutions of the same stock solution. On average, the total signals observed varied by ≈6% in both optical systems. Because the total signal is generally difficult to measure due to signal offsets from traces of rapidly sedimenting aggregates, as well as very slowly sedimenting small species and baseline offsets, we also studied the signal amplitude of only the BSA monomer species, which was hydrodynamically resolved and baseline separated in all data sets included in the present study. There was no discernable anti-correlation of monomer and dimer signal in the absorbance data, as expected for hydrodynamically well-resolved and well-defined species (**S9 Fig**). The interference data were broadly similar in this respect, although some data sets showed high dimer fractions usually associated with tilting plateaus and narrow fit ranges (see above). As mentioned above, there is also no discernable trend of BSA monomer signal magnitude with time (**S4 Fig**). Therefore, the BSA monomer signal was considered the best marker for relative signal magnification in different instruments.

**Fig 11:**
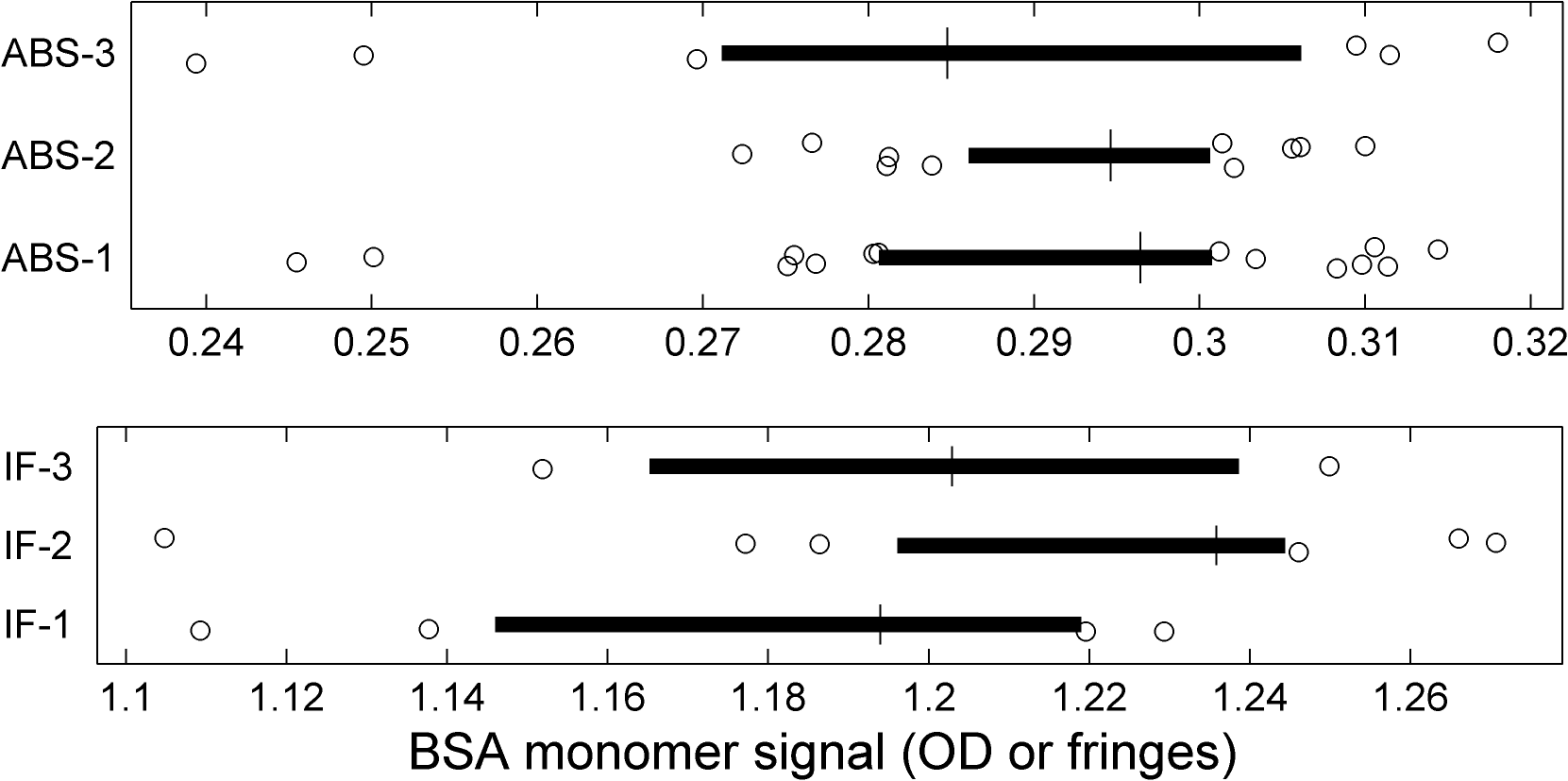
Distributions of calculated BSA monomer signals for the different kits and the different optical systems. The box-and-whisker plots indicate the central 50% of the data as solid line and draw the smaller and larger 25% percentiles as individual circles. The median for each group is displayed as vertical line.

The statistics of the total signal amplitude of the BSA monomer, as calculated by integration of all baseline-resolved monomer peaks in *c*(*s*), is shown in **Fig 11**for absorbance and interference data for the different kits (which may vary slightly due to pipetting errors). For the absorbance data, the standard deviation (relative to the kit-average) is 5.6%, similar to the value of the total signal. This is far above the statistical error of ≈0.3% of the monomer signal we obtained in the analysis of a SV data set as a result of errors from the stochastic noise in the data acquisition.

An important consideration in this context is a variation in the data acquisition wavelength due to a limited precision of the monochromator control. While the scans were set up to take readings at 280 nm, the actual wavelengths reported in the scan files ranged from 278 nm to 282 nm, with an average of 279.8 nm ± 1.0 nm. No correlation of the monomer signal with the reported wavelength was apparent (**S10 Fig**). However, errors in the wavelength calibration of each instrument would contribute to the errors in the absorbance readings. Other instrumental factors contributing to errors in absorbance readings in the AUC include stray light, and possibly small variations in cell alignment or flash timing.

For the fringe shift amplitude of the BSA monomer determined with the interference optics, we found a variation of 3.6%, which may be related to limited precision in the measurement of the underlying pixel-per-fringe value which is specific for each instrument dependent on optical alignment. (Three instruments were equipped with a 675 nm laser, as opposed to the currently used 655 nm laser, which causes only a ≈0.28% calculated difference in the value of *dn/dc*, based on [40], which was neglected in the consideration of signal magnitudes.) Thus, an instrument-dependent error of at least ≈3–4% in concentration should be considered when determining protein concentrations based on an assumed refractive index increment and given fringe shift data (in addition to variations in the protein refractive index increments, which may be significant especially for smaller proteins [41]).

Related, a time-honored approach to experimentally determine a protein extinction coefficient is based on the ratio of the absorbance signal magnitude and the refractive index signal. For all instruments for which both absorbance scans reported at 280 nm and interference data at 655 nm were available, variations in both optical signals were uncorrelated (**S11 Fig**), and exhibited a standard deviation of 4.8%. Thus, this value appears to be a lower limit for the accuracy by which absolute extinction coefficients can be measured in AUC using the dual-signal method. (This does not apply to relative extinction coefficients when carrying out multi-signal experiments in a single instrument.)

### Fraction of trace dimer

**Fig 12:**
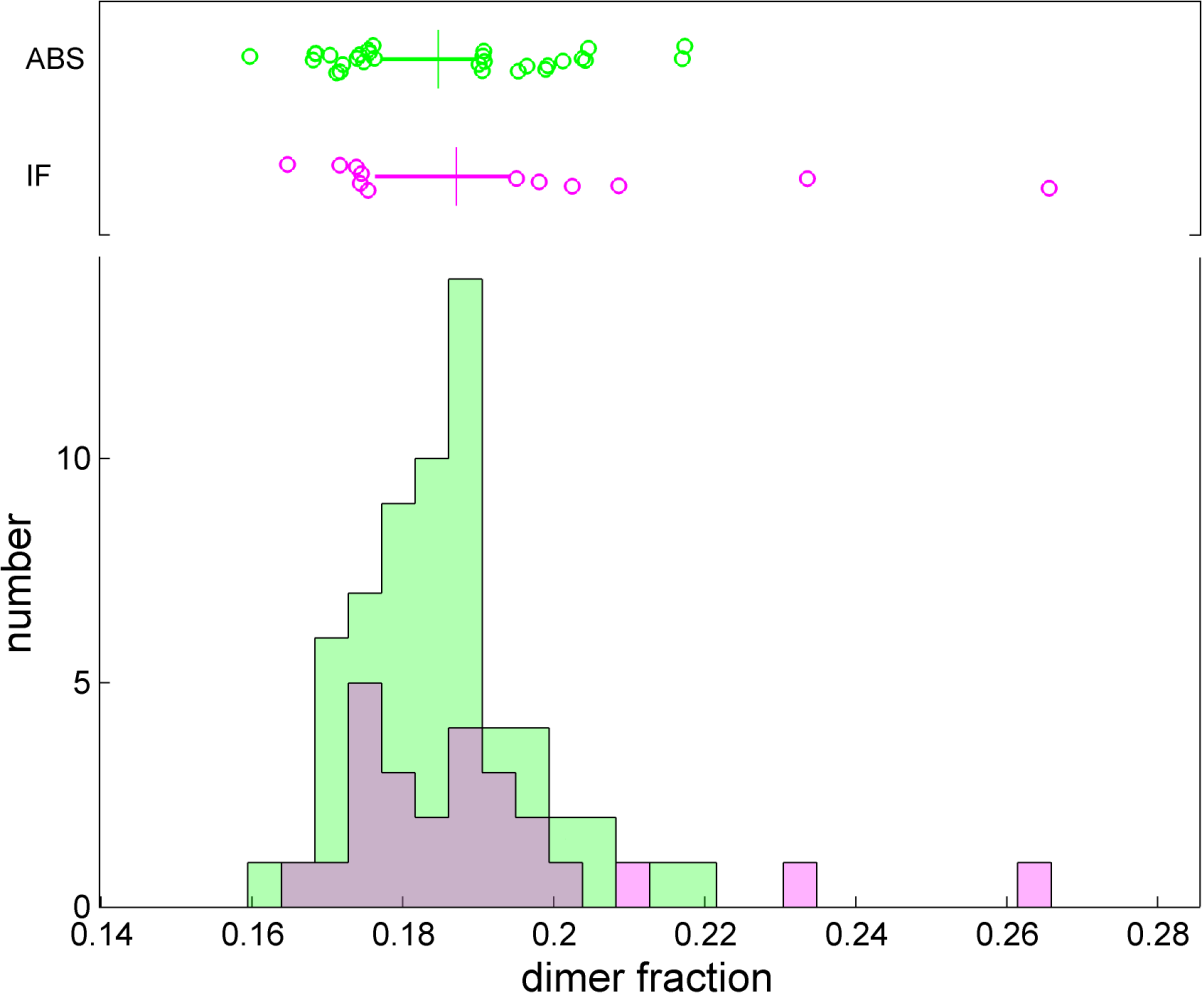
Observed fraction of dimer (as a ratio of dimer peak area to the sum of monomer plus dimer peak areas). The box-and-whisker plot indicates the central 50% of the data as solid line and draws the smaller and larger 25% percentiles as individual circles. The median displayed as vertical line. The mean and standard deviations are 18.5% ± 1.1% for the absorbance system, and 19.0% ± 2.1% for the interference system.

A quantity of interest in many biotechnology laboratories is the determination of the dimer fraction of a protein sample. The BSA sample provided an opportunity to test the instrument-to-instrument variation of this parameter, for data where the dimer peak is resolved (**Fig 12**). From the absorbance data, the dimer fraction was 18.5% ± 1.1%, and from the interference data it was 19.0% ± 2.1% (or 18.6% ± 1.5% if the two extreme values are excluded). However, a limitation in this assessment is the possibility for a slight trend in the dimer fraction, due to the duration of the study, possibly decreasing by less than 2% per year (**S5 Fig**, see above).

### BSA monomer apparent molar mass and frictional coefficient

**Fig 13:**
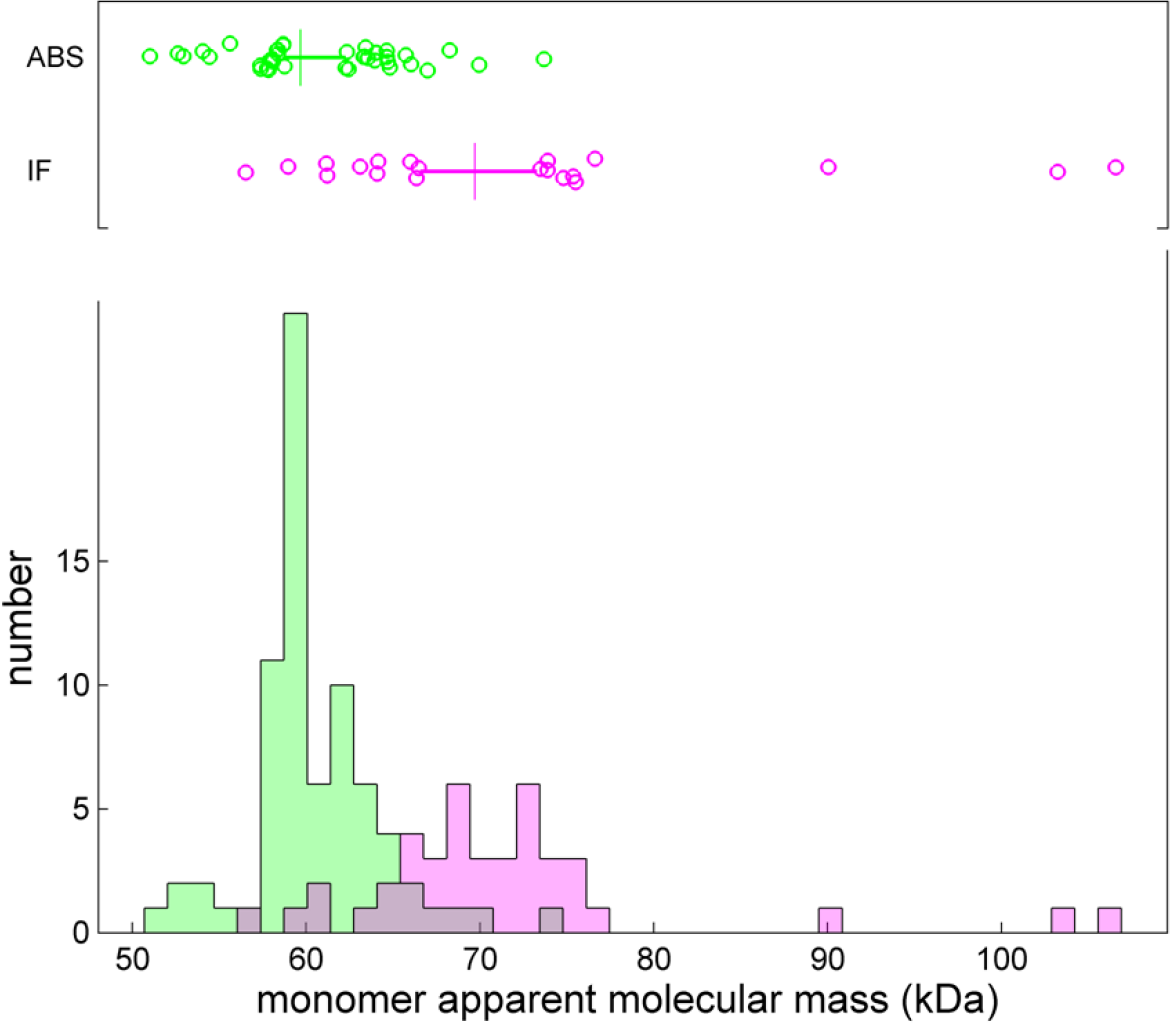
Corrected best-fit apparent monomer molecular mass from integration of the *c*(*s*) peak when scanned with the absorbance system (green) and the interference system (magenta). Only data with rmsd less than 0.01 OD or 0.01 fringes were included. The box-and-whisker plot indicates the central 50% of the data as solid line and draws the smaller and larger 25% percentiles as individual circles. The median is displayed as a vertical line.

Finally, it is of interest to study how reproducibly the parameters characterizing the boundary broadening can be determined. For this, we only considered fits that led to an rmsd value of less than 0.01 OD or 0.01 fringes, respectively. The apparent molar mass measured by the absorbance and interference system was (60.6 ± 3.2) kg/mol and (70.5 ± 8.1) kg/mol, respectively. Despite the close correspondence of the *s*_*20T*,*t*,*r*,*v*_-values with (4.328 ± 0.025) S and (4.317 ± 0.035) S for the absorbance and interference system, respectively, surprisingly, there is a clear difference between their apparent molar mass values (**Fig 13**). It originates from shallower measured boundaries in the absorbance system as compared to the interference system, which resulted in uncorrected best-fit frictional ratios of 1.29 ± 0.05 and 1.44 ± 0.13, respectively. The magnitude of this difference in broadening is far greater than the calculated impact of finite scan speed in the absorbance system on apparent boundary broadening, which for the current scan settings and *s*-value would be negligible [36]. The origin of the difference is unclear, although one could speculate that it may be related to differences in the optical adjustment, such as the focal point, leading to differences in the radial resolution of the two optical systems, and/or non-linear signal distortions of the boundary from stray light [19].

**Fig 14:**
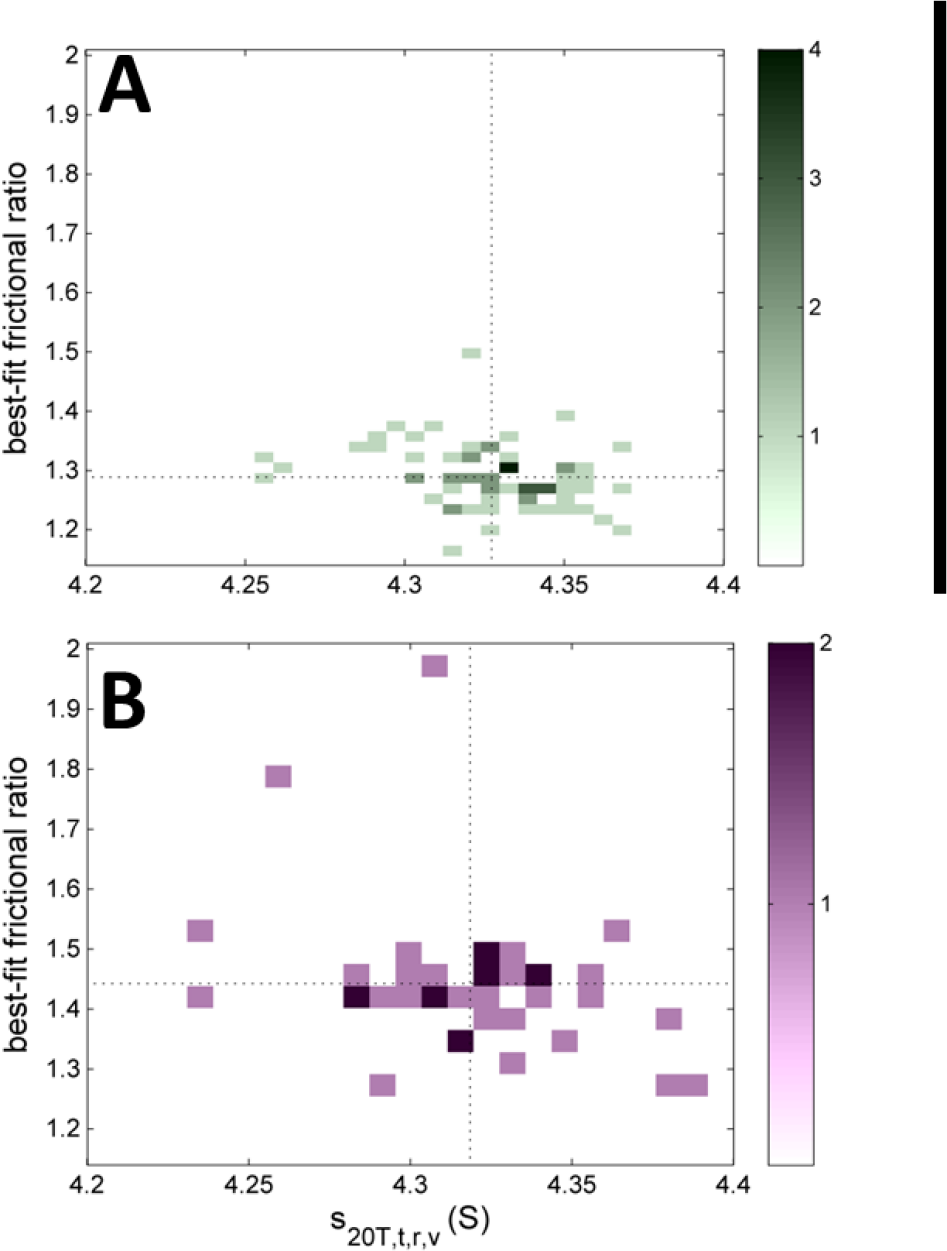
Correlation between the best-fit frictional ratio from the *c*(*s*) analysis and the final *s*_*20T*,*t*,*r*,*v*_-value for the absorbance system (A) and interference system (B). Data are shown as a histograms with frequency values indicated in the colorbars. The dotted lines indicate the average frictional ratio and *s*_***20T*,*t*,*r*,*v***_-values of both systems, respectively. To aid the comparison, both histograms are plotted in the same scale.

Neither the average molar mass from integration of the *c*(*s*) monomer peak (not shown) nor the underlying best-fit signal average frictional ratio from the *c*(*s*) analysis (**Fig 14**) exhibited a strong correlation with the corrected sedimentation coefficients *s*_*20T*,*t*,*r*,*v*_. A slight anti-correlation of frictional ratio and *s*_*20T*,*t*,*r*,*v*_-values may be discerned within the spread of values, especially in the data from the absorbance system (**Fig 14A**). This pattern that would be consistent with convection being one of the parameters underlying the residual variation of *s*_*20T*,*t*,*r*,*v*_-values, but the experimental data suggest that this is at least not the dominant source of residual variation of sedimentation coefficients.

## Conclusions

The large-scale side-by-side comparison of data obtained for the same sample in different instruments offers unique insights in many technical aspects of AUC that are highly relevant for the detailed interpretation of AUC experiments, which is essential in many traditional and advanced current applications of AUC.

The present study has clearly shown that external calibration of time, radial magnification, and rotor temperature are each essential for the quantitative interpretation of results from the AUC, confirming and expanding the results of a previous small pilot study [27]. Without external controls, *s*-values for the BSA monomer were measured from 3.7 S to 4.9 S, a range spanning ±15% with a standard deviation of 4.4%. Importantly, none of these data sets gave obvious indications of instrumental malfunction or operator errors. Furthermore, it must be kept in mind that the deviations are not subject to significant run-to-run variation on the same instrument. Even though the comparison of many instruments makes the deviations appear stochastic, for a given instrument they are systematic, i.e. the results will be very similar in repeat experiments. For example, even though it would be an outlier in the total ensemble, an instrument leading to *s*-values 10% below the true value—which may occur from errors in either temperature, radial magnification or scan time—will consistently produce such data, without giving any indication of malfunction or inferior performance and therefore likely unbeknownst to the researchers using this instrument, until rare adventitious events might inadvertently change calibration settings. In fact, without calibration corrections, 57% of all measured s-values in the present study fell outside the two standard deviation confidence level of the mean after corrections.

The present study also clearly demonstrates that an effective way to address this problem is to carry out the complete set of external calibration procedures, which cover most data dimensions, and to monitor the sedimentation parameters of a stable reference sample. The calibration references, consisting of intervals of file creation time-stamp for time, a radial reference mask, and iButton temperature loggers for temperature measurement in the spinning or resting rotor, are easy to measure, and can improve the quantitative accuracy to a level adequate for most current data interpretation. The residual error has a standard deviation of 0.7% and is consistent with commonly assumed accuracy, and slightly better, for example, than historically assessed for the Spinco Model E [24]. After corrections, only 7% of s-values are outside the two standard deviation confidence range.

As a test for consistency of the calibration measurements we found BSA in PBS to be an excellent standard. Although we have not looked into batch-to-batch variation, it has historically been used in many studies for this purpose, with reports of very similar *s*-values (e.g., 4.29 S in [29], and 4.32 S in [24]). In conjunction with modern *c*(*s*) analysis it is possible to focus on the monomer, independent of possible slow aggregation processes. However, despite the long time at which the samples were kept mostly at ambient temperature, and the repeated stress on the protein sample from temperature fluctuations during shipping, we found it to be extremely stable over the course of nearly a year. Thus, it is suitable as a convenient long-term reference to test repeatability and accuracy in individual laboratories, if kept in a sealed cell assembly.

A data dimension that was not covered here is the accuracy of the rotor speed. Even though the parameters responsible for the remaining variation of the *s*_*20T*,*t*,*r*,*v*_-values are unclear, on the basis of the data from the present study the rotor speed can be excluded from being a major source of instrument-to-instrument variation. This is consistent with recent measurements in a limited number of instruments that found the accuracy of rotor speed to be on the order of 0.01% [27]. Rather, we believe the remaining spread of *s*_*20T*,*t*,*r*,*v*_-values to be a result of residual calibration errors in radius and temperature, and of temperature- and/or radial alignment-driven convection in SV experiments.

We have previously estimated the accuracy of measurement of the rotor temperature with iButtons to be 0.2°C or better [27,33]. With the temperature-dependence of the viscosity of water, this would translate to a limit in the accuracy of *s*-values of ≈0.5%. This is consistent with the residual standard deviation of 0.7% of the *s*_*20T*,*t*,*r*,*v*_-values of the BSA monomer after all external calibration corrections. Conversely, if the BSA monomer *s*-value is taken as a marker for temperature, under the unrealistic assumption all *s*-value errors be caused by temperature-driven viscosity differences, then the remaining error of 0.7% in the *s*_*20T*,*t*,*r*,*v*_-values would limit to ≈0.3°C the possible remaining stochastic error of the temperature measurements. These results clearly demonstrate a substantial improvement in accuracy when using the iButton as compared to the use of the instrument internal radiometer readings for determining the relevant solvent viscosity. In the present study the iButton approach was for the first time used in large number of AUC laboratories, showing its robustness and ease-of-use.

While the current study was ongoing, we have found that iButtons can also be used on the resting rotor, as described in [34]. Accordingly, the protocol was expanded for most remaining laboratories to include a comparison of the temperature measurement in the spinning and resting rotor. A third approach for the iButton measurements, with the location in a modified rotor handle to provide data in real-time during the high-speed SV, is clearly the most advantageous, but could obviously not be included in the present study for prohibitive cost and logistical difficulties of shipping a rotor. Even though based on a smaller data set, the results support the validity of the resting rotor approach. It has the obvious advantage of not requiring any custom modification of the rotor handle [33], and not requiring a custom inset to fit the iButton into the barrel of the cell assembly, such that it can be easily carried out in any AUC laboratory.

As expected, the time error of the scan files was strongly reduced from the ≈10% error in data acquisition software 6.0 and its associated firmware [31] due to a recent update, although a cohort of 9 instruments were still subject to this error (and two instruments exhibited an error by 1000%). Most instrument data showed time errors between 0.12% to 0.3%. This is still a significant systematic error (and larger errors can be encountered at different scan settings from those used in the present protocol [27]). Fortunately, it can be entirely avoided and conveniently removed by replacing the file header time with operating system time stamps [27,31]. This approach was incorporated and is enabled by default in SEDFIT.

Radial calibration errors are also highly relevant, and become immediately obvious in the comparison of *s*-values from data acquired side-by-side with the absorbance and interference system, where all temperature-related and time-related factors are canceled out. The good correlation between the ratio of *s*-values and the ratio of radial magnification correction factors (**Fig 4**) independently supports the application of the radial calibration mask. Unfortunately, such a mask is currently not widely available without custom fabrication. This is currently being addressed in an ongoing collaboration between laboratories of the U.S. National Institute of Biomedical Imaging and Bioengineering and the U.S. National Institute of Standards and Technology (NIST), aimed at the development of an improved radial reference standard for AUC.

In order to implement the conclusions from the present study in the routine practice in AUC laboratories, it would seem ideal if complete reference kits could be assembled and stored locally in each laboratory. Much like those in the present multi-laboratory study, they could consist of one sealed cell assembly with BSA sample, one sealed cell assembly with a radial reference mask, as well as an iButton for temperature measurements. We propose that as a matter of routine maintenance by the investigators, the calibration experiments could then be carried out on a regular schedule, and additionally after instrument service or repair. This would enable tracking of any changes in instrument performance, and allow determination of current calibration correction factors to ensure good quantitative accuracy of the data.

## Supporting Information

S1 Fig Long-term stability of iButton temperature readings. Temperature readings as a function of experiment time for the three kits (blue, green, and magenta circles and solid lines). Highlighted as bold solid line is the console set point of 20°C. Also shown are side-by-side measurements of the three iButtons in the same instrument (not included in the study) at two different points in time (magenta and blue squares and green star, dotted lines). Reproducibility of the iButtons in repeat experiments is better than 0.06°C [27].
(TIF)

S2 Fig Long-term stability of the radial mask. (A) Shown are radial magnification correction factors as a function of time of experiment for the three kits (blue, green, and magenta). Highlighted as black line and grey patch are the mean and standard deviation of the values after excluding the three largest outliers. (B) The bottom plot is an expanded view of (A) excluding the outliers.
(TIF)

S3 Fig Long-term stability of the cell assembly. Shown are the best-fit meniscus positions for the three kits (blue, green, and magenta) as a function of time. Highlighted as horizontal solid lines and patches are the mean and standard deviations of the values for each kit. A slight drift may be discerned in the data from the kit presented in blue, with a slope corresponding to a sample volume change at a rate of approximately 5 μL/year.
(TIF)

S4 Fig Long-term trend in the monomer signal. Calculated monomer signal in the absorbance optics (A) and interference optics (B) for the three kits (blue, green, and magenta) as a function of time. The results are obtained by integration of the *c*(*s*) monomer peak, excluding data where peaks are not well separated. Highlighted as horizontal solid line and grey patch are the mean and standard deviations.
(TIF)

S5 Fig Long-term trend in the dimer fraction. Calculated dimer fraction in the absorbance optics (circles and solid lines) and interference optics (crosses and dotted lines) for the three kits (blue, green, and magenta). The values are calculated as the ratio of the integrals of the *c*(*s*) monomer and dimer peaks, and exclude data where peaks are not well separated. Highlighted as horizontal solid line and grey patch are the mean and standard deviation.
(TIF)

S6 Fig Indicators of convection as a function of initial temperature jump. Correlations of the initial transient temperature jump during the SV experiment and the *s*_*20T*,*t*,*r*,*v*_-values of the BSA monomer (A) and the ratio of the rmsd in the 1mm of data points closest to the meniscus to the overall rmsd (B). Data are shown as a histograms with frequency values indicated in the colorbars.
(TIF)

S7 Fig Example for a data set with likely initial convection. Screenshot of SEDFIT window for analysis of data set with initial convection, as indicated by the misfit in the highlighted region. The overall rmsd was 0.0047 OD, but higher by a factor 1.11 within the 1 mm of the lower fitting limit.
(TIF)

S8 Fig Example for a data set with sloping plateaus. Screenshot of SEDFIT window for analysis of an interference data set with sloping plateaus in the region highlighted in red.
(TIF)

S9 Fig Correlation between monomer and dimer signal. Data are shown for the absorbance data (A) and interference data (B) as a histograms with frequency values indicated in the colorbars. The solid line represents a perfect proportionality of both values, whereas the dotted line represents a perfect anti-correlation of both signals at constant total signal.
(TIF)

S10 Fig Absence of correlation between monomer signal and nominal acquisition wavelength. Histogram of the deviation of the BSA monomer absorbance signal from their kit average values, plotted as a function of reported absorbance wavelength, with frequency values as indicated in the colorbar.
(TIF)

S11 Fig Correlation between absorbance and interference signals. Histogram of the absorbance signal of the BSA monomer, as determined from the integration of the *c*(*s*) monomer peak, and the interference signal from the same run and the same instrument. Frequency values are as indicated in the colorbar. A similar correlation plot lacking apparent correlations is obtained in a corresponding plot of differences of monomer signals to the kit averages (data not shown).
(TIF)

S1 Table Data underlying the statistical analyses. (PDF)
