# Supplementary figures and images for: A Multilaboratory Comparison of Calibration Accuracy and the Performance of External References in Analytical Ultracentrifugation
Source: PLoS One. 2015 May 21;10(5):e0126420. doi: 10.1371/journal.pone.0126420 (PMC4440767; doi:10.1371/journal.pone.0126420)

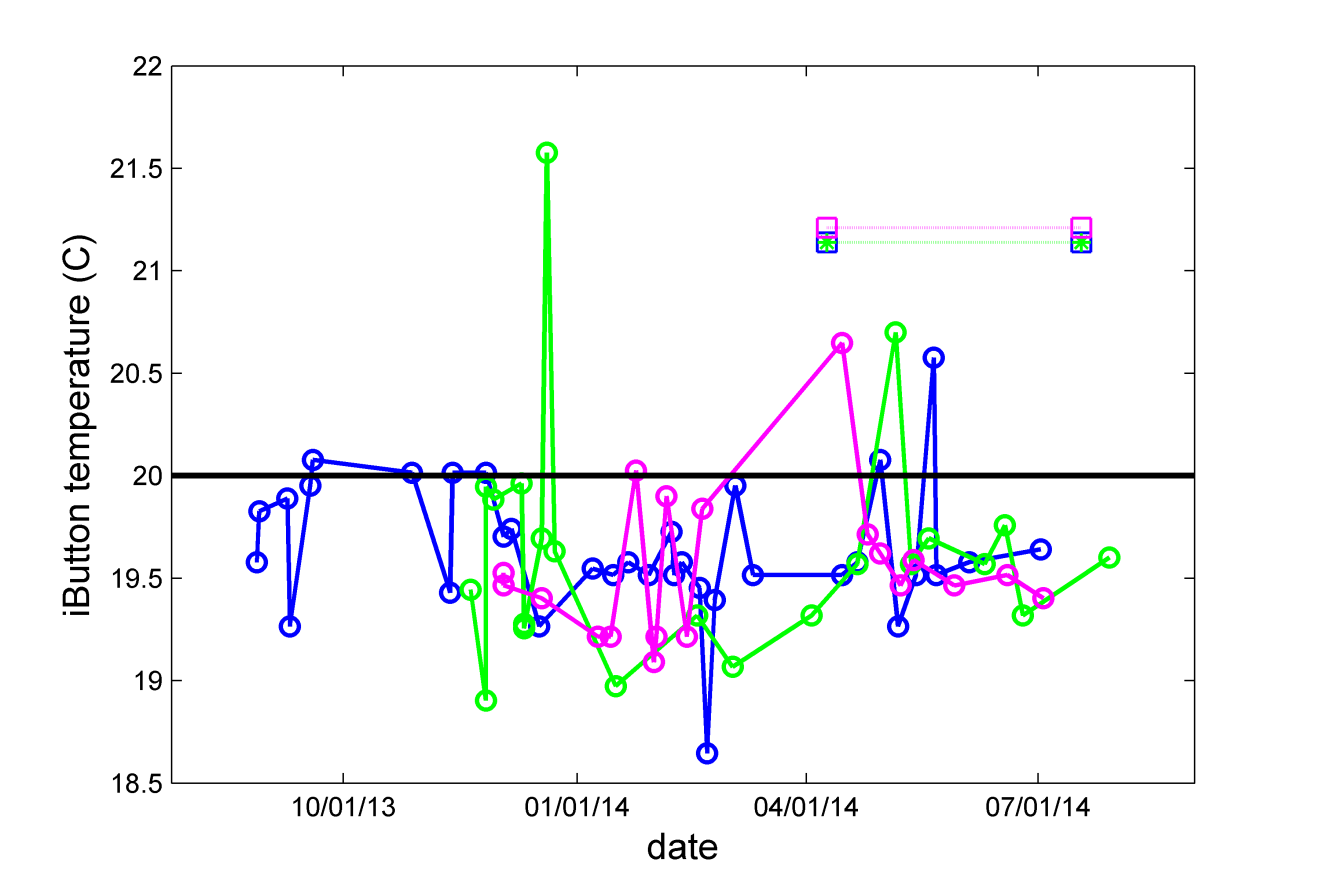

Supplement: S1 Fig — Temperature readings as a function of experiment time for the three kits (blue, green, and magenta circles and solid lines). Highlighted as bold solid line is the console set point of 20°C. Also shown are side-by-side measurements of the three iButtons in the same instrument (not included in the study) at two different points in time (magenta and blue squares and green star, dotted lines). Reproducibility of the iButtons in repeat experiments is better than 0.06°C [27]. (TIF) [file pone.0126420.s001.tif]

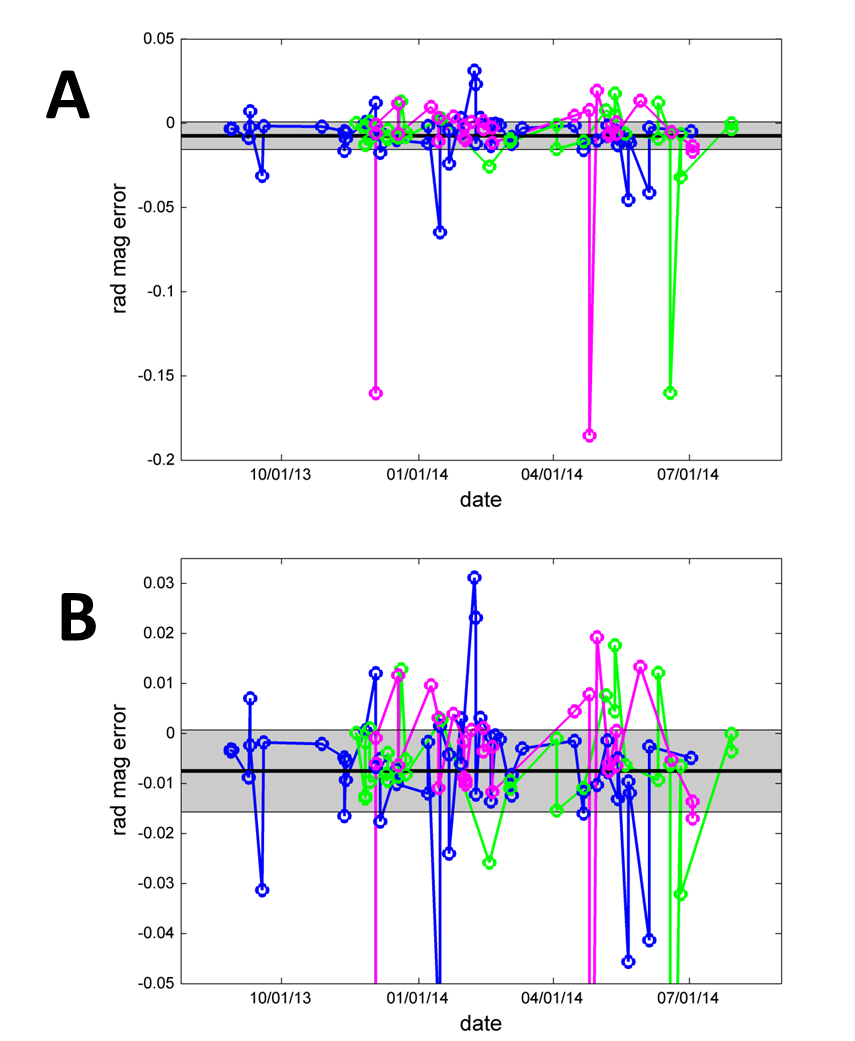

Supplement: S2 Fig — (A) Shown are radial magnification correction factors as a function of time of experiment for the three kits (blue, green, and magenta). Highlighted as black line and grey patch are the mean and standard deviation of the values after excluding the three largest outliers. (B) The bottom plot is an expanded view of (A) excluding the outliers. (TIF) [file pone.0126420.s002.tif]

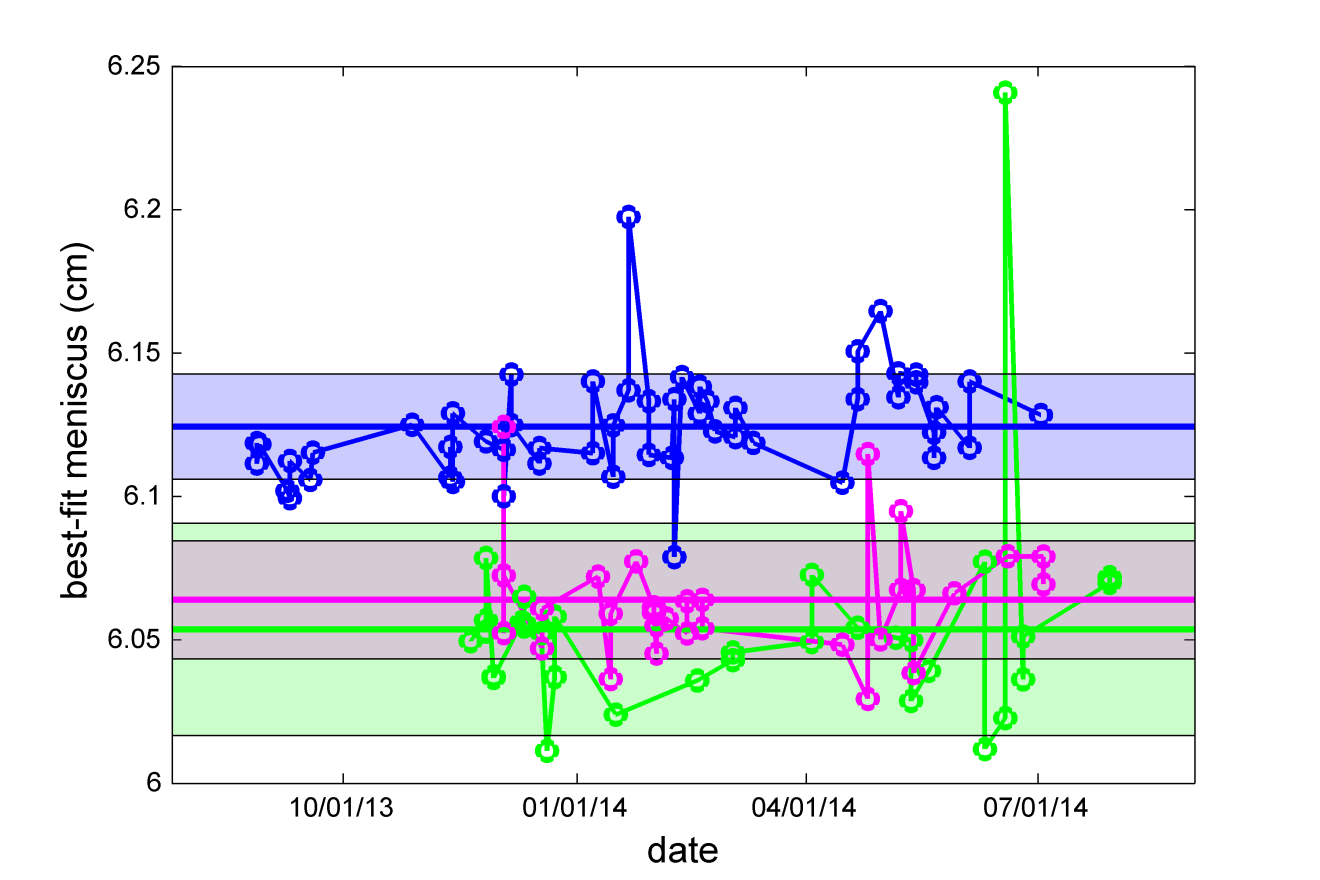

Supplement: S3 Fig — Shown are the best-fit meniscus positions for the three kits (blue, green, and magenta) as a function of time. Highlighted as horizontal solid lines and patches are the mean and standard deviations of the values for each kit. A slight drift may be discerned in the data from the kit presented in blue, with a slope corresponding to a sample volume change at a rate of approximately 5 μL/year. (TIF) [file pone.0126420.s003.tif]

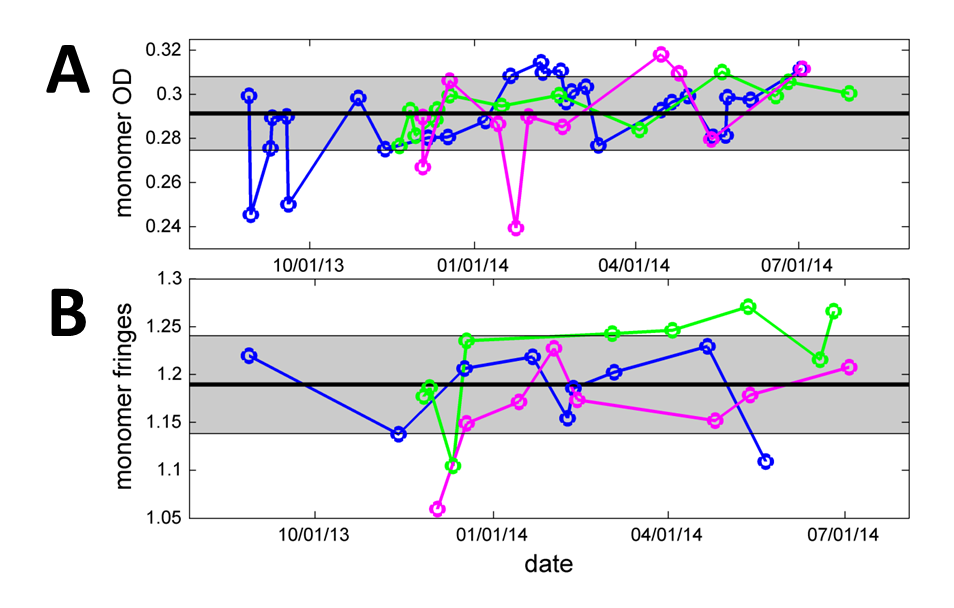

Supplement: S4 Fig — Calculated monomer signal in the absorbance optics (A) and interference optics (B) for the three kits (blue, green, and magenta) as a function of time. The results are obtained by integration of the c(s) monomer peak, excluding data where peaks are not well separated. Highlighted as horizontal solid line and grey patch are the mean and standard deviations. (TIF) [file pone.0126420.s004.tif]

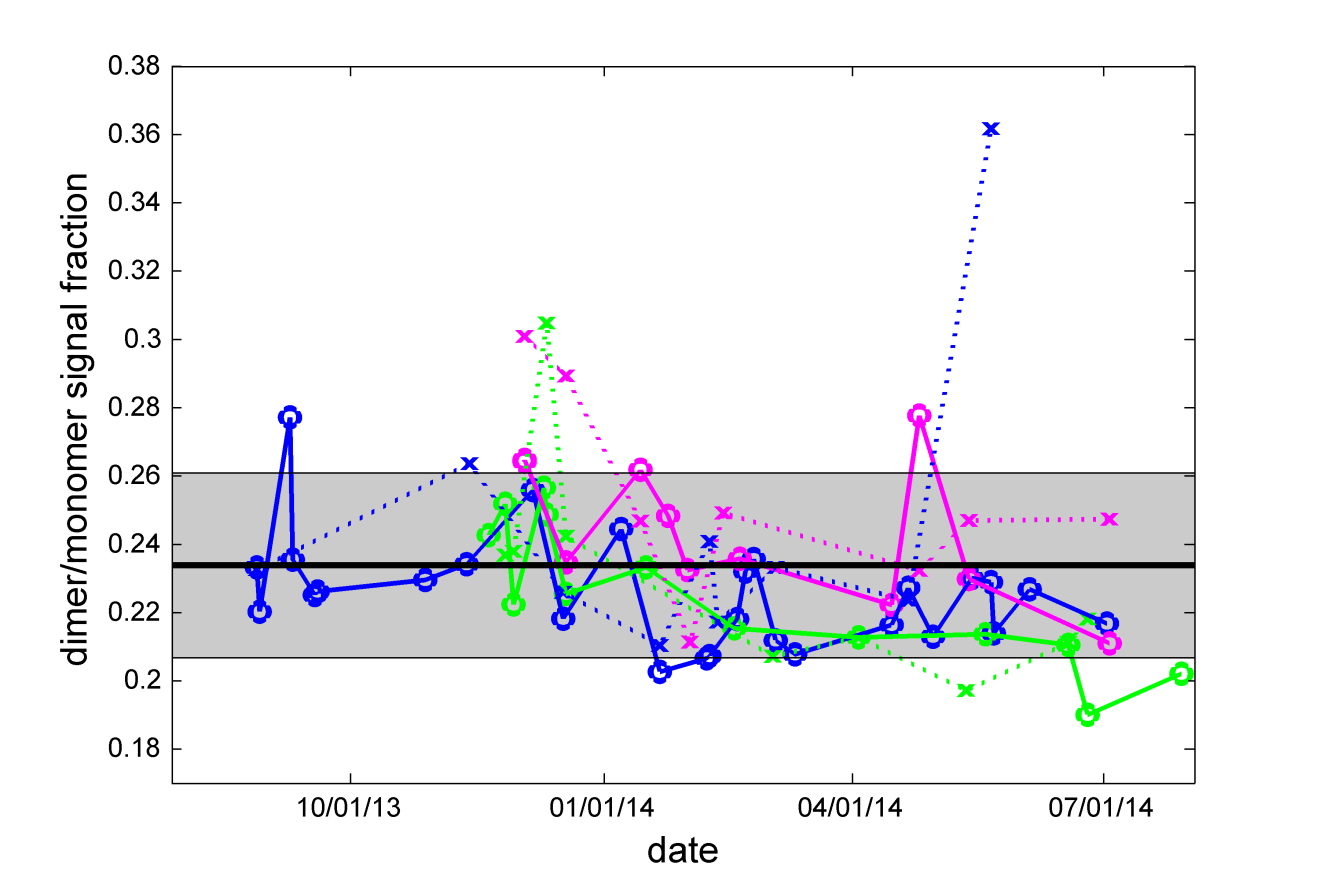

Supplement: S5 Fig — Calculated dimer fraction in the absorbance optics (circles and solid lines) and interference optics (crosses and dotted lines) for the three kits (blue, green, and magenta). The values are calculated as the ratio of the integrals of the c(s) monomer and dimer peaks, and exclude data where peaks are not well separated. Highlighted as horizontal solid line and grey patch are the mean and standard deviation. (TIF) [file pone.0126420.s005.tif]

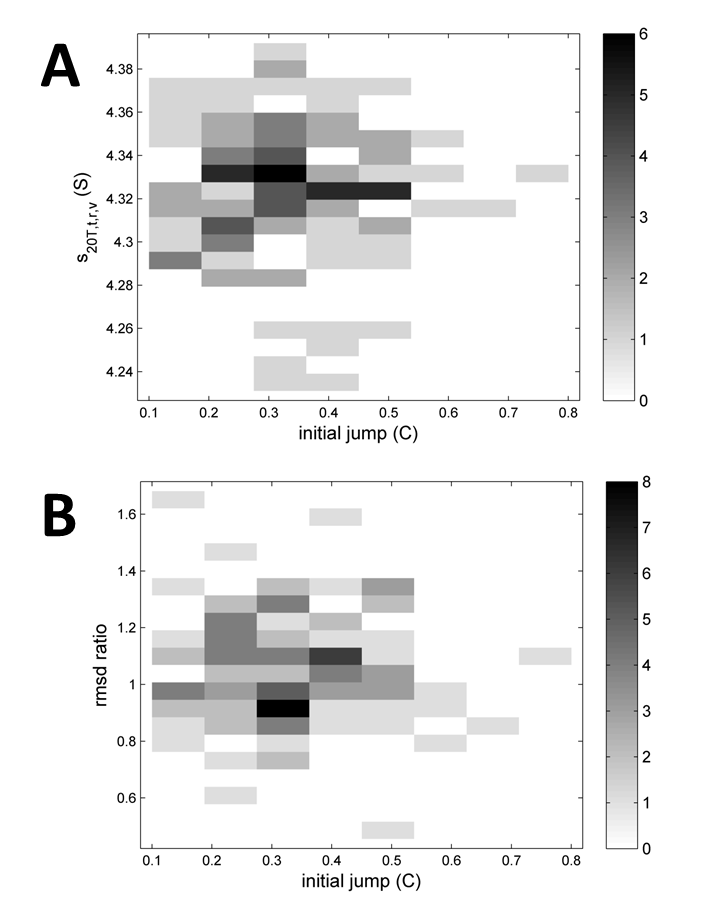

Supplement: S6 Fig — Correlations of the initial transient temperature jump during the SV experiment and the s 20T,t,r,v-values of the BSA monomer (A) and the ratio of the rmsd in the 1mm of data points closest to the meniscus to the overall rmsd (B). Data are shown as a histograms with frequency values indicated in the colorbars. (TIF) [file pone.0126420.s006.tif]

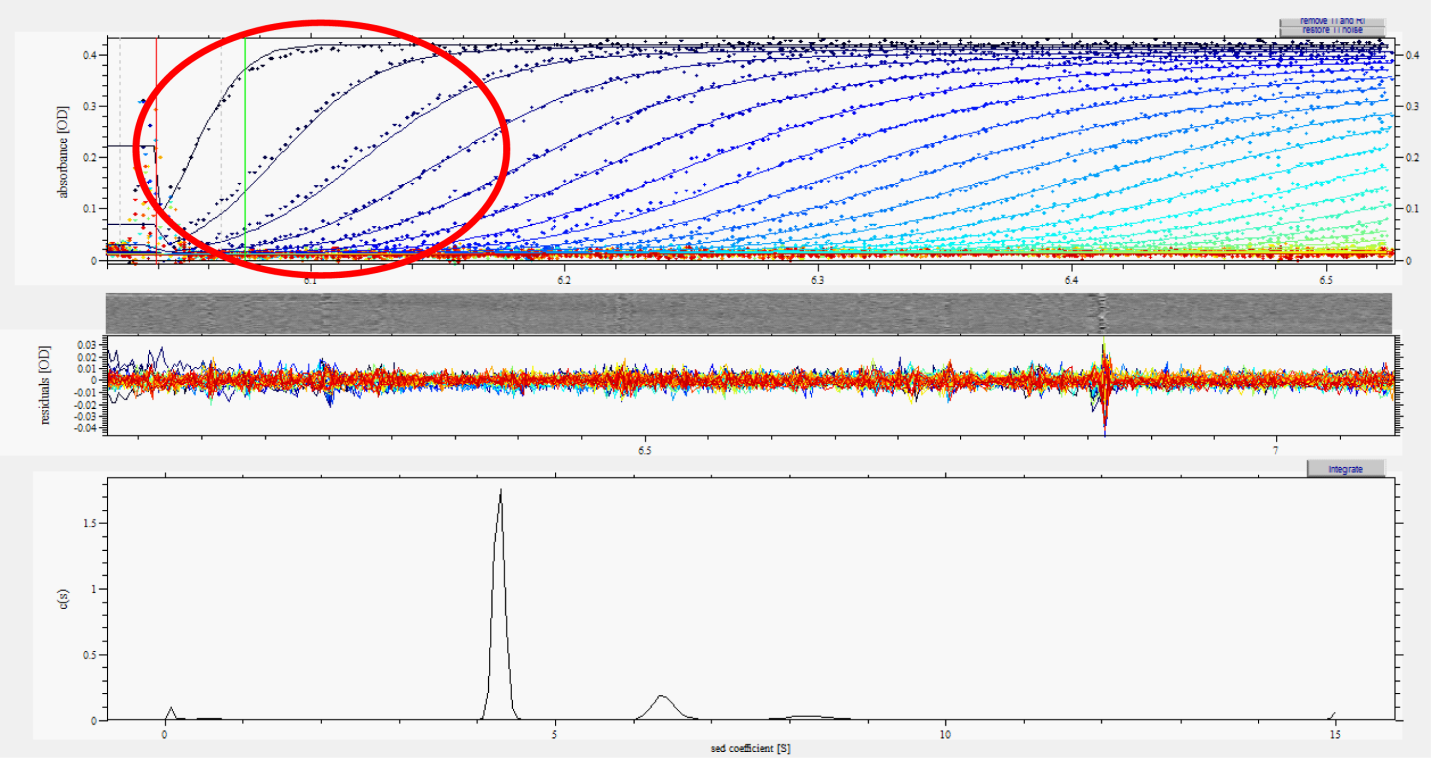

Supplement: S7 Fig — Screenshot of SEDFIT window for analysis of data set with initial convection, as indicated by the misfit in the highlighted region. The overall rmsd was 0.0047 OD, but higher by a factor 1.11 within the 1 mm of the lower fitting limit. (TIF) [file pone.0126420.s007.tif]

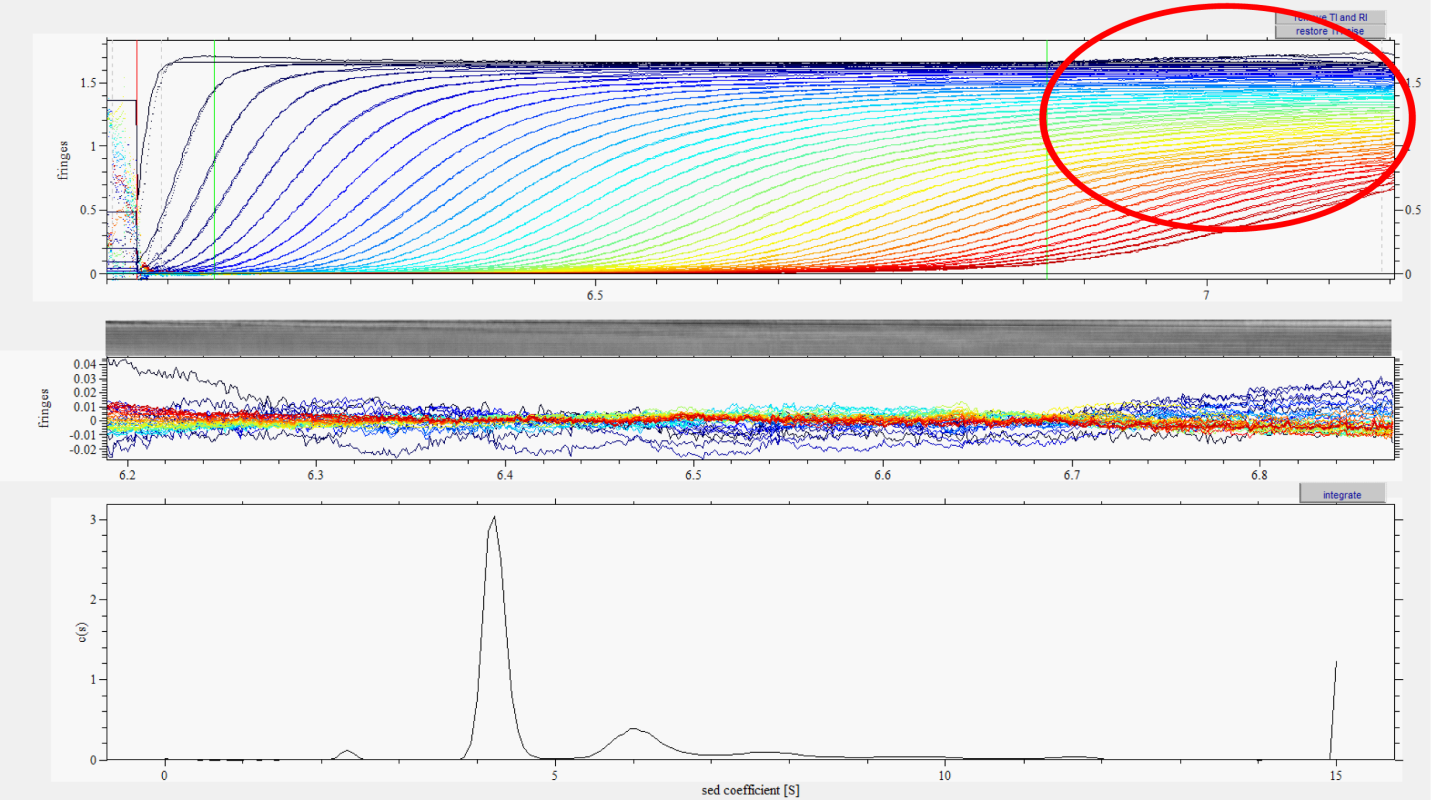

Supplement: S8 Fig — Screenshot of SEDFIT window for analysis of an interference data set with sloping plateaus in the region highlighted in red. (TIF) [file pone.0126420.s008.tif]

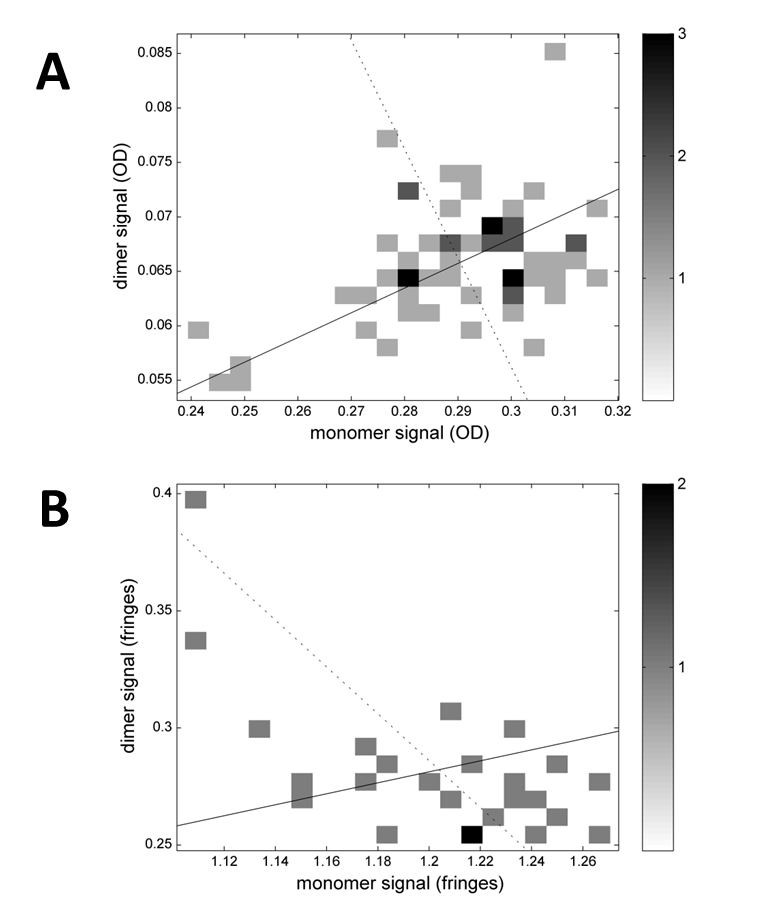

Supplement: S9 Fig — Data are shown for the absorbance data (A) and interference data (B) as a histograms with frequency values indicated in the colorbars. The solid line represents a perfect proportionality of both values, whereas the dotted line represents a perfect anti-correlation of both signals at constant total signal. (TIF) [file pone.0126420.s009.tif]

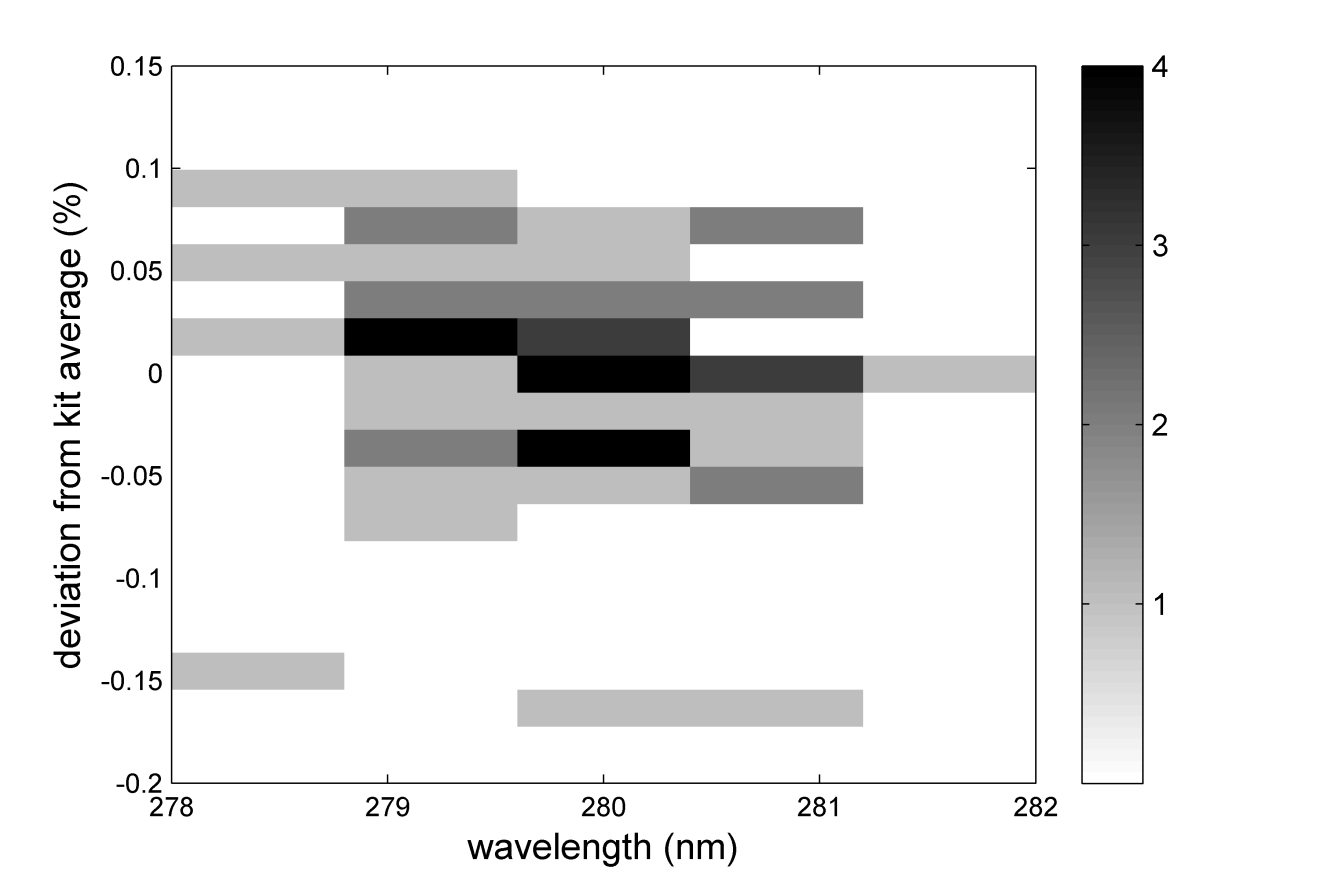

Supplement: S10 Fig — Histogram of the deviation of the BSA monomer absorbance signal from their kit average values, plotted as a function of reported absorbance wavelength, with frequency values as indicated in the colorbar. (TIF) [file pone.0126420.s010.tif]

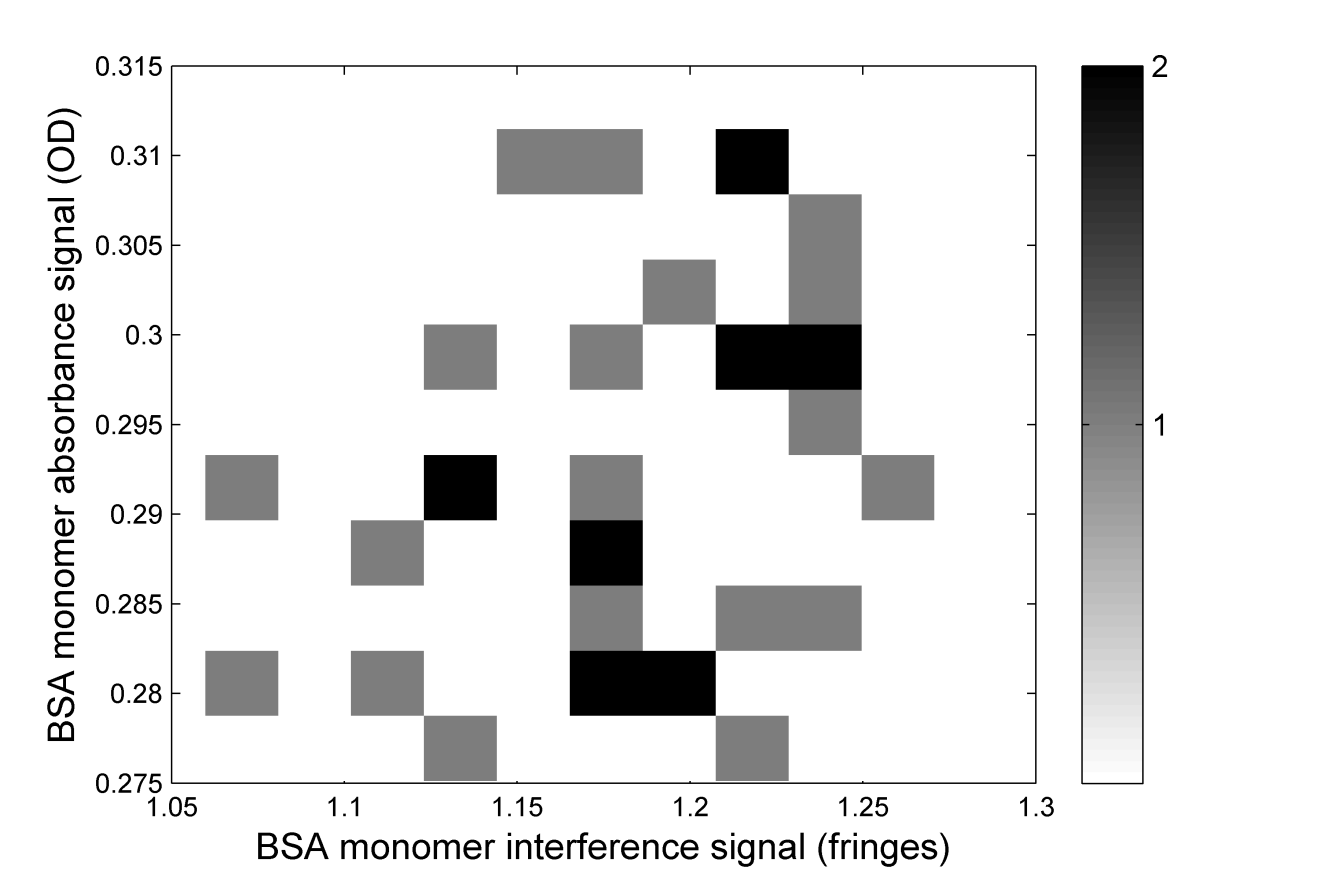

Supplement: S11 Fig — Histogram of the absorbance signal of the BSA monomer, as determined from the integration of the c(s) monomer peak, and the interference signal from the same run and the same instrument. Frequency values are as indicated in the colorbar. A similar correlation plot lacking apparent correlations is obtained in a corresponding plot of differences of monomer signals to the kit averages (data not shown). (TIF) [file pone.0126420.s011.tif]
