# Supplementary material for: A Multilaboratory Comparison of Calibration Accuracy and the Performance of External References in Analytical Ultracentrifugation
Source: PLoS One. 2015 May 21;10(5):e0126420. doi: 10.1371/journal.pone.0126420 (PMC4440767; doi:10.1371/journal.pone.0126420)
Supplement: S1 Table — (PDF) [file pone.0126420.s012.pdf]

kit No.	AUC type	optics	iButton temp (°C)	run temp (°C)	max temp jump (°C)	T-factor (%)	offset T-factor	time error	scan velocity error	R-factor	sraw (S)	s20,t (S)	s20,T (S)	s20,r (S)	s20t,T,r,v (S)	f/f0	rmsd	monomer signal	monomer signal%	MW of monomer (kDa)
2	XLI	IF	19.7580	20.0	0.3	-0.60%		0.13%	0.00%	-16.00%	3.655	3.651	3.677	4.240	4.260	1.79	0.007	1.215	67.05%	77.61
3	XLI	IF	19.7138	20.0	0.3	-0.71%		0.13%	0.00%	-18.53%	3.656	3.651	3.682	4.334	4.358	1.93	0.011	1.152	68.86%	87.07
3	XLI	IF	19.5269	20.0	0.4	-1.17%		0.14%	0.00%	-16.02%	3.677	3.672	3.720	4.266	4.310	1.99	0.006	1.060	64.82%	91.84
1	XIL	ABS	19.5156	20.0	0.3	-1.20%		0.13%	0.18%	-6.48%	4.020	4.015	4.069	4.281	4.318	1.51	0.005	0.277	67.94%	69.19
2	XLI	IF	19.3185	20.0	0.2	-1.69%		0.13%	0.00%	-3.21%	4.108	4.103	4.177	4.240	4.306	1.42	0.009	1.266	61.77%	65.30
1	XLI	ABS	19.5780	20.2	0.2	-1.04%	-0.55%	0.15%	0.18%	-4.13%	4.121	4.115	4.143	4.291	4.300	1.37	0.004	0.297	71.29%	62.06
1	XLI	IF	19.5780	20.0	0.1	-1.04%		0.13%	0.00%	-2.40%	4.152	4.147	4.196	4.252	4.291	1.25	0.008	1.218	75.06%	55.20
3	XLI	IF	19.4023	20.0	0.4	-1.48%		0.12%	0.00%	-1.70%	4.173	4.168	4.235	4.244	4.302	1.46	0.005	1.208	70.86%	69.77
1	XLI	IF	19.4531	20.0	0.2	-1.36%		0.15%	0.00%	-1.36%	4.179	4.173	4.236	4.236	4.287	1.40	0.006	1.173	63.14%	65.85
2	XLI	IF	18.9037	20.0	0.5	-2.75%		0.28%	0.00%	-1.30%	4.179	4.167	4.294	4.233	4.338	1.47	0.002	1.177	70.24%	70.30
3	XLI	ABS		20.0	0.5			0.14%	0.18%	-1.77%	4.183	4.177				1.27	0.007	0.250	74.95%	56.55
1	XLI	IF	19.2658	20.0	0.5	-1.83%		0.13%	0.00%	-0.77%	4.187	4.181	4.263	4.219	4.290	1.40	0.005	1.124	68.48%	66.06
3	XLI	IF	19.0906	20.1	0.1	-2.27%	-2.02%	0.25%	0.00%	-0.88%	4.187	4.177	4.272	4.224	4.298	1.49	0.008	1.140	65.50%	72.57
2	XLI	IF	19.3185	20.0	0.2	-1.69%		0.14%	0.00%	-1.54%	4.190	4.184	4.261	4.255	4.321	1.39	0.003	1.246	74.33%	65.33
1	XLI	ABS	20.5770	20.0	0.3	1.40%		0.13%	0.18%	-4.56%	4.194	4.188	4.135	4.385	4.310	1.37	0.003	0.281	70.46%	64.06
1	XLI	ABS	18.6457	20.0	0.3	-3.41%		0.14%	0.18%	-0.04%	4.200	4.194	4.343	4.201	4.331	1.30	0.006	0.296	72.27%	59.32
2	XLI	ABS	18.9037	20.0	0.5	-2.75%		0.27%	0.18%	-1.25%	4.200	4.189	4.315	4.252	4.349	1.39	0.005	0.293	67.39%	65.21
1	XLI	ABS	19.5780	20.0	0.2	-1.04%		0.13%	0.18%	-1.60%	4.203	4.198	4.247	4.271	4.302	1.32	0.004	0.296	64.33%	60.99
3	XLI	ABS	19.4023	20.0	0.4	-1.48%		0.13%	0.18%	-1.36%	4.207	4.202	4.269	4.264	4.314	1.33	0.006	0.311	75.74%	61.37
1	XLI	ABS	19.5156	20.0	0.5	-1.20%		0.14%	0.18%	-1.31%	4.212	4.206	4.262	4.267	4.304	1.35	0.006	0.281	71.20%	63.19
1	XLI	IF	19.5780	20.0	0.2	-1.04%		0.13%	0.00%	-1.17%	4.213	4.207	4.257	4.262	4.300	1.42	0.007	1.229	72.42%	68.12
3	XLI	ABS	19.2153	20.0	0.3	-1.95%		0.17%	0.18%	-1.02%	4.217	4.210	4.299	4.260	4.328	1.34	0.004	0.284	73.28%	62.66
1	XLI	IF	19.2658	20.0	0.4	-1.83%		0.14%	0.00%	-1.01%	4.218	4.212	4.295	4.261	4.332	1.39	0.003	1.206	72.16%	65.81
2	XLI	IF	19.0674	20.0	0.4	-2.33%		0.26%	0.00%	-1.10%	4.219	4.208	4.317	4.266	4.354	1.41	0.004	1.243	65.60%	67.08
1	XLI	ABS	19.2658	20.0	0.4	-1.83%		0.14%	0.18%	-0.72%	4.220	4.214	4.297	4.251	4.314	1.28	0.005	0.281	71.22%	58.34
2	XLI	IF	19.2776	20.0	0.2	-1.80%		0.15%	0.00%	-0.94%	4.222	4.216	4.298	4.262	4.332	1.48	0.005	1.105	65.45%	72.58
3	XLI	IF	19.2153	20.0	0.3	-1.95%		0.16%	0.00%	-0.94%	4.225	4.218	4.307	4.265	4.341	1.41	0.004	1.227	73.30%	67.60
1	XLA	ABS	19.5156	20.0	0.3	-1.20%		0.12%	0.18%	-1.19%	4.226	4.221	4.277	4.276	4.314	1.29	0.003	0.299	72.87%	59.13
1	XLI	IF	19.7410	20.1	0.4	-0.64%		0.13%	0.00%	-1.76%	4.231	4.225	4.258	4.305	4.327	1.44	0.006	1.067	63.88%	69.87

3	XLI	IF	19.2153	20.0	0.5	-1.95%		0.15%	0.00%	-0.36%	4.231	4.225	4.314	4.247	4.323	1.49	0.005	1.174	69.23%	73.21
2	XLI	ABS	19.0674	20.0	0.3	-2.33%		0.27%	0.18%	-0.95%	4.232	4.220	4.330	4.272	4.352	1.29	0.005	0.306	76.82%	58.85
1	XLI	IF	19.2658	20.0	0.3	-0.18%		0.30%	0.00%	-0.24%	4.233	4.220	4.240	4.243	4.238	1.51	0.006	1.112	68.35%	75.18
3	XLI	IF		20.0	0.5			0.13%	0.00%	-0.62%	4.233	4.228				1.34	0.003	1.249	72.55%	62.71
3	XLI	IF	19.4023	20.0	0.3	-1.48%		0.15%	0.00%	-0.64%	4.237	4.231	4.300	4.264	4.321	1.51	0.009	1.149	67.77%	75.01
1	XLI	IF	19.5156	20.0	0.5	-1.20%		0.13%	0.00%	-0.50%	4.238	4.232	4.289	4.259	4.305	1.47	0.005	1.183	70.21%	72.21
2	XLA	ABS	19.2558	20.0	0.3	-1.85%		0.20%	0.18%	-0.39%	4.239	4.231	4.318	4.256	4.319	1.29	0.004	0.287	75.23%	59.28
2	XLI	ABS	19.2776	20.0	0.1	-1.80%		0.15%	0.18%	-0.95%	4.241	4.234	4.317	4.281	4.344	1.26	0.006	0.293	70.85%	57.53
3	XLI	IF	19.4646	20.0	0.5	-1.33%		0.13%	0.00%	-0.75%	4.241	4.235	4.297	4.273	4.324	1.47	0.004	1.155	68.88%	72.41
1	XLI	IF	19.5156	20.0	0.3	-1.20%		0.14%	0.00%	0.15%	4.249	4.243	4.300	4.242	4.287	1.45	0.007	1.140	65.97%	70.75
1	XLI	ABS	19.5468	20.0	0.3	-1.12%		0.27%	0.18%	-1.20%	4.252	4.241	4.300	4.303	4.332	1.35	0.006	0.288	70.23%	64.04
1	XLI	ABS	19.2658	20.0	0.5	-1.83%		0.13%	0.18%	-0.14%	4.253	4.247	4.330	4.259	4.323	1.43	0.021	0.299	58.61%	69.84
1	XLI	IF	19.7029	20.0	0.1	-0.73%	-0.39%	0.27%	0.00%	-0.67%	4.257	4.245	4.273	4.285	4.290	1.48	0.011	1.069	61.21%	73.39
3	XLI	ABS	19.2153	20.0	0.4	-1.95%		0.15%	0.18%	0.11%	4.257	4.250	4.340	4.252	4.321	1.34	0.006	0.285	71.80%	63.49
1	XLI	IF	19.5156	20.0	0.5	-1.20%		0.28%	0.00%	-0.61%	4.259	4.247	4.310	4.285	4.324	1.43	0.008	1.169	70.34%	69.48
1	XLI	IF	19.5780	20.2	0.2	-1.04%	-0.55%	0.15%	0.00%	-0.26%	4.259	4.253	4.282	4.270	4.287	1.42	0.004	1.174	69.41%	68.74
2	XLI	ABS	19.5696	20.0	0.3	-1.06%		0.27%	0.18%	-0.93%	4.261	4.249	4.306	4.300	4.327	1.28	0.006	0.302	75.86%	58.67
1	XLI	IF	19.5780	20.0	0.3	-1.04%		0.15%	0.00%	-0.36%	4.261	4.255	4.306	4.277	4.315	1.35	0.004	1.220	62.67%	63.91
2	XLI	ABS	19.3185	20.0	0.3	-1.69%		0.13%	0.18%	-0.10%	4.265	4.259	4.337	4.269	4.327	1.28	0.003	0.284	76.15%	59.53
1	XLI	ABS	19.5780	20.0	0.1	-1.04%		0.13%	0.18%	-0.42%	4.265	4.259	4.309	4.282	4.314	1.16	0.005	0.308	75.70%	50.80
2	XLA	ABS	19.9631	20.0	0.1	-0.09%		0.17%	0.18%	-0.81%	4.265	4.258	4.269	4.300	4.289	1.33	0.006	0.288	69.42%	62.84
1	XLI	IF	19.5468	20.0	0.3	-1.12%		0.27%	0.00%	-0.17%	4.267	4.255	4.314	4.274	4.310	1.42	0.007	1.169	69.11%	69.37
3	XLI	ABS	19.0906	20.1	0.2	-2.27%	-2.02%	0.27%	0.18%	-0.16%	4.267	4.255	4.353	4.274	4.340	1.27	0.004	0.290	71.68%	58.28
1	XLI	IF	20.5770	20.0	0.3	1.40%		0.15%	0.00%	-0.96%	4.268	4.262	4.209	4.310	4.243	1.27	0.012	1.109	66.94%	58.40
3	XLI	IF	19.8385	20.0	0.2	-0.40%		0.11%	0.00%	-1.17%	4.268	4.264	4.285	4.318	4.331	1.46	0.004	1.143	71.91%	71.98
2	XLI	ABS	19.7580	20.0	0.2	-0.60%		0.13%	0.18%	-0.63%	4.271	4.266	4.297	4.298	4.310	1.26	0.005	0.299	75.95%	57.71
2	XLA	ABS	19.6325	20.0	0.4	-0.91%		0.14%	0.18%	-0.52%	4.274	4.268	4.313	4.296	4.321	1.29	0.005	0.304	75.91%	60.22
3	XLI	ABS	19.5892	20.0	0.5	-1.02%		0.15%	0.18%	0.05%	4.274	4.268	4.318	4.272	4.301	1.28	0.004	0.279	72.57%	59.34
2	XLA	ABS	19.4441	20.0	0.5	-1.38%		0.12%	0.18%	0.01%	4.276	4.271	4.335	4.275	4.322	1.31	0.009	0.277	71.39%	61.82
2	XLI	ABS	19.3185	20.0	0.2	-1.69%		0.14%	0.18%	-0.65%	4.277	4.272	4.350	4.305	4.364	1.17	0.028	0.306	66.96%	52.28
1	XLI	IF	20.0151	20.0	0.8	0.04%		0.14%	0.00%	-0.72%	4.278	4.272	4.276	4.309	4.301	1.40	0.005	1.162	68.60%	67.77
3	XLI	IF	19.5892	20.0	0.5	-1.02%		0.15%	0.00%	-0.64%	4.278	4.272	4.321	4.305	4.343	1.45	0.006	1.179	68.71%	71.69
2	XLA	ABS	18.9733	20.0	0.2	-2.57%		0.22%	0.18%	0.27%	4.278	4.269	4.388	4.267	4.359	1.31	0.006	0.295	74.93%	61.66
2	XLI	IF	19.6011	20.0	0.3	-0.99%		0.15%	0.00%	-0.36%	4.281	4.275	4.323	4.296	4.332	1.33	0.006	1.240	72.31%	62.87

1	XLI	IF	19.5780	20.0	0.2	-1.04%		0.13%	0.00%	0.31%	4.283	4.277	4.327	4.269	4.308	1.48	0.010	1.186	72.88%	74.11
2	XLA	ABS	19.6952	20.0	0.4	-0.75%		0.18%	0.18%	-0.63%	4.284	4.276	4.316	4.311	4.327	1.29	0.005	0.310	74.78%	60.10
1	XLI	IF	19.9527	20.0	0.2	-0.12%		0.26%	0.00%	-0.83%	4.290	4.279	4.295	4.326	4.319	1.36	0.003	1.202	73.01%	65.44
1	XLI	ABS	19.5780	20.0	0.3	-1.04%		0.14%	0.18%	-0.32%	4.291	4.285	4.336	4.305	4.336	1.26	0.005	0.299	73.02%	58.67
1	XLA	ABS	19.5156	20.0	0.3	-1.20%		0.13%	0.18%	-0.15%	4.292	4.286	4.343	4.298	4.336	1.26	0.005	0.293	73.03%	58.28
2	XLI	IF	19.6952	20.0	0.3	-0.75%		0.14%	0.00%	-0.66%	4.292	4.286	4.324	4.320	4.347	1.33	0.004	1.235	68.06%	63.66
1	XLA	ABS	19.5156	20.0	0.3	-1.20%		0.14%	0.18%	-0.30%	4.293	4.287	4.345	4.306	4.344	1.27	0.005	0.277	72.02%	58.81
1	XLI	ABS	19.7410	20.1	0.3	-0.64%		0.13%	0.18%	-0.51%	4.293	4.288	4.321	4.316	4.330	1.30	0.006	0.280	68.87%	61.06
2	XLI	ABS	19.6952	20.0	0.3	-0.75%		0.13%	0.18%	-0.86%	4.295	4.289	4.327	4.332	4.351	1.30	0.004	0.300	75.44%	61.47
2	XLI	ABS	19.6011	20.0	0.3	-0.99%		0.14%	0.18%	-0.01%	4.297	4.291	4.339	4.297	4.326	1.26	0.006	0.300	77.50%	58.31
3	XLI	ABS	19.9008	20.0	0.2	-0.24%		0.11%	0.18%	0.07%	4.298	4.293	4.309	4.295	4.293	1.36	0.006	0.280	69.71%	65.77
1	XLI	ABS	19.4531	20.0	0.2	-1.36%		0.14%	0.18%	-0.07%	4.298	4.292	4.357	4.301	4.346	1.26	0.008	0.311	71.85%	58.64
1	XLI	IF	19.5156	20.2	0.3	-1.20%	-0.70%	0.14%	0.00%	-1.22%	4.299	4.293	4.329	4.351	4.376	1.38	0.007	1.154	71.80%	67.27
3	XLI	ABS	19.4646	20.3	0.4	-1.33%	-0.58%	0.13%	0.18%	1.33%	4.299	4.293	4.324	4.242	4.253	1.33	0.008	0.273	68.16%	63.27
1	XLI	ABS	19.9527	20.0	0.2	-0.12%		0.27%	0.18%	-1.24%	4.299	4.288	4.304	4.352	4.338	1.27	0.006	0.303	72.24%	58.94
3	XLI	ABS	19.4646	20.0	0.5	-1.33%		0.13%	0.18%	-0.60%	4.300	4.295	4.357	4.326	4.370	1.26	0.015	0.293	71.06%	58.72
1	XLI	ABS	19.2658	20.0	0.4	-0.18%		0.29%	0.18%	0.70%	4.300	4.288	4.308	4.270	4.258	1.28	0.005	0.289	72.94%	59.93
3	XLA	ABS	19.4646	20.0	0.5	-1.33%		0.20%	0.18%	-0.09%	4.305	4.296	4.362	4.309	4.349	1.31	0.006	0.267	68.56%	61.96
3	XLI	ABS	19.2153	20.0	0.4	-1.95%		0.27%	0.18%	0.96%	4.307	4.295	4.391	4.265	4.329	1.27	0.006	0.291	71.52%	59.45
3	XLI	ABS	19.5269	20.0	0.3	-1.17%		0.14%	0.18%	-0.60%	4.307	4.301	4.358	4.333	4.370	1.27	0.006	0.290	70.31%	59.63
1	XLI	ABS	19.6404	20.0	0.2	-0.89%		0.13%	0.18%	-0.49%	4.308	4.302	4.346	4.329	4.354	1.27	0.003	0.311	72.50%	59.42
1	XLA	ABS	19.3936	20.3	0.6	-1.50%	-0.76%	0.13%	0.18%	-0.12%	4.308	4.303	4.340	4.313	4.332	1.31	0.005	0.301	70.39%	62.26
2	XLA	ABS	19.6325	20.0	0.2	-0.91%		0.11%	0.18%	-0.82%	4.309	4.304	4.348	4.344	4.371	1.33	0.005	0.283	73.95%	64.11
1	XLI	ABS	19.5156	20.0	0.5	-1.20%		0.27%	0.18%	0.30%	4.316	4.304	4.368	4.303	4.335	1.19	0.015	0.281	70.42%	54.21
3	XLI	ABS	19.4023	20.0	0.6	-1.48%		0.15%	0.18%	1.16%	4.317	4.311	4.381	4.267	4.316	1.27	0.006	0.306	73.55%	59.51
3	XLA	ABS	20.0255	20.0	0.3	0.06%		0.11%	0.18%	0.39%	4.318	4.313	4.315	4.301	4.286	1.34	0.007	0.239	60.37%	64.94
1	XLA	ABS	20.0151	20.0	0.1	0.04%		0.14%	0.18%	-0.21%	4.320	4.314	4.318	4.329	4.314	1.24	0.005	0.298	73.18%	57.26
3	XLI	ABS	19.8385	20.0	0.2	-0.40%		0.12%	0.18%	-0.27%	4.322	4.317	4.340	4.334	4.338	1.24	0.009	0.285	74.16%	57.59
3	XLI	ABS	19.5156	20.3	0.4	-1.20%	-0.45%	0.23%	0.18%	-0.53%	4.325	4.315	4.344	4.348	4.350	1.26	0.007	0.271	72.37%	59.13
2	XLI	ABS	19.5696	20.0	0.4	-1.06%		0.15%	0.18%	0.44%	4.339	4.332	4.385	4.320	4.351	1.25	0.004	0.293	76.30%	58.92
1	XLI	ABS	20.0775	20.2	0.2	0.19%	0.67%	0.15%	0.18%	-0.18%	4.341	4.335	4.312	4.349	4.306	1.28	0.004	0.250	73.09%	60.84
2	XLI	ABS	19.9463	20.0	0.6	-0.13%		0.16%	0.18%	-0.18%	4.352	4.345	4.358	4.360	4.351	1.24	0.006	0.281	74.07%	57.81
1	XLI	ABS	20.0151	20.0	0.8	0.04%		0.14%	0.18%	0.06%	4.353	4.348	4.352	4.351	4.335	1.32	0.006	0.291	69.96%	63.58
3	XLI	ABS	19.7138	20.0	0.3	-0.71%		0.12%	0.18%	0.78%	4.357	4.352	4.388	4.323	4.341	1.26	0.008	0.309	73.99%	59.50

1	XLI	ABS	19.7029	20.0	0.1	-0.73%	-0.39%	0.27%	0.18%	1.20%	4.381	4.369	4.398	4.328	4.325	1.24	0.008	0.292	68.42%	60.08
3	XLA	ABS	16.6204	20.0	0.2	-0.94%		0.12%	0.18%	1.92%	4.384	4.379	4.425	4.300	4.328	1.19	0.005	0.270	70.85%	55.50
2	XLI	IF	19.5696	20.0	0.3	-1.06%		0.28%	0.00%	1.21%	4.395	4.383	4.442	4.342	4.376	1.28	0.004	1.237	59.01%	61.95
3	XLI	ABS	20.6486	20.1	0.4	1.57%	1.33%	0.14%	0.18%	0.44%	4.413	4.407	4.355	4.394	4.321	1.23	0.005	0.318	74.58%	59.01
1	XLA	ABS	19.7275	20.0	0.4	-0.67%		0.15%	0.18%	3.11%	4.416	4.409	4.446	4.279	4.293	1.17	0.017	0.314	69.72%	54.64
2	XLI	IF	19.5696	20.0	0.3	-1.06%		0.14%	0.00%	1.76%	4.430	4.424	4.477	4.352	4.392	1.27	0.008	1.271	71.91%	62.25
1	XLI	ABS	19.5156	20.2	0.4	-1.20%	-0.70%	0.13%	0.18%	2.31%	4.457	4.452	4.489	4.354	4.371	1.19	0.005	0.310	73.19%	56.93
2	XLA	ABS	21.5775	19.9	0.4	3.74%		0.13%	0.18%	1.28%	4.606	4.600	4.434	4.547	4.364	1.22	0.005	0.299	73.84%	61.67
1	XLI-2	IF	19.4302	20.0	0.2	-1.41%		10.12%	0.00%	-1.65%	4.612	4.188	4.677	4.688	4.317	1.42	0.005	1.208	62.47%	67.17
1	XLA	ABS	19.9527	20.0	0.2	-0.12%		10.69%	0.18%	-3.13%	4.648	4.199	4.653	4.794	4.328	1.34	0.004	0.290	73.30%	62.00
2	XLA	ABS	19.3185	20.0	0.1	-1.69%		11.14%	0.18%	-2.58%	4.651	4.185	4.730	4.771	4.358	1.29	0.005	0.300	75.56%	58.37
3	XLI	IF	19.2153	20.0	0.1	-1.95%		10.22%	0.00%	-1.09%	4.664	4.232	4.755	4.715	4.361	1.52	0.006	1.171	68.05%	75.78
1	XLI-1	IF	20.0151	20.5	0.4	0.04%	1.25%	9.90%	0.00%	-0.93%	4.665	4.245	4.607	4.709	4.231	1.40	0.005	1.138	69.09%	67.70
1	XLI-2	ABS	19.4302	20.0	0.2	-1.41%		10.12%	0.18%	-0.48%	4.690	4.259	4.756	4.713	4.332	1.30	0.007	0.275	61.01%	60.72
2	XLA	ABS	19.5696	20.0	0.1	-1.06%		10.77%	0.18%	-1.09%	4.692	4.236	4.742	4.743	4.320	1.31	0.004	0.272	74.55%	61.12
2	XLI	IF	19.8835	20.0	0.3	-0.29%		9.88%	0.00%	-0.97%	4.726	4.301	4.739	4.771	4.355	1.47	0.008	1.186	71.52%	74.07
1	XLI-1	ABS	20.0151	20.5	0.5	0.04%	1.25%	9.92%	0.18%	-0.55%	4.728	4.301	4.669	4.754	4.263	1.31	0.006	0.292	73.01%	61.93
3	XLI	ABS	19.2153	20.0	0.1	-1.95%		10.23%	0.18%	0.31%	4.747	4.307	4.840	4.733	4.370	1.28	0.017	0.287	71.87%	60.10
1	XLA	ABS	20.0775	20.0	0.7	0.19%		10.85%	0.18%	-1.03%	4.753	4.288	4.744	4.802	4.316	1.24	0.005	0.299	73.19%	56.78
2	XLI	ABS	19.8835	20.0	0.3	-0.29%		9.87%	0.18%	0.10%	4.757	4.330	4.771	4.752	4.330	1.26	0.003	0.281	75.04%	59.49
2	XLA	ABS	20.6993	20.0	0.4	1.69%		10.69%	0.18%	0.76%	4.949	4.471	4.865	4.912	4.355	1.23	0.007	0.301	75.04%	59.85
1	XLA	ABS	19.8902	20.0	0.1	-0.27%		901.69%	0.18%	-0.88%	42.702	4.263	42.817	43.078	4.304	1.40	0.018	0.275	65.15%	67.65
1	XLA	ABS	19.8277	20.0	0.2	-0.41%		901.79%	0.18%	-0.33%	43.287	4.321	43.465	43.432	4.345	1.24	0.010	0.245	75.52%	57.64
1	XLI	ABS	19.5780	20.0	0.2	-1.04%		0.13%	0.18%	0.73%						1.26	0.114	0.347	53.93%	61.25
2	XLI	IF	19.9463	20.0	0.6	-0.13%		0.14%	0.00%			4.245				1.38	0.006	1.206	71.31%	65.67
2		FDS	19.5169			-1.20%														
2	XLA	ABS	19.5069			-1.22%														
2			19.5069																	
2			19.3948																	
3	XLI	IF	19.2153			-1.95%		0.26%	0.00%											
3	XLI	IF	19.9008			-0.24%			0.00%			4.355				1.36	0.007	1.250	70.73%	
3	XLI	IF	20.6486	20.1	0.4	1.57%	1.33%	0.13%	0.00%	0.15%										
